# Confounder-adjusted MRI-based predictors of multiple sclerosis disability

**DOI:** 10.3389/fradi.2022.971157

**Published:** 2022-09-13

**Authors:** Yujin Kim, Mihael Varosanec, Peter Kosa, Bibiana Bielekova

**Affiliations:** Laboratory of Clinical Immunology and Microbiology, Neuroimmunological Diseases Section, National Institutes of Health, National Institute of Allergy and Infectious Diseases, Bethesda, MD, United States

**Keywords:** magnetic resonance imaging, physiological confounders, multiple sclerosis, disability outcomes, machine learning, gradient boosting machine

## Abstract

**Introduction:**

Both aging and multiple sclerosis (MS) cause central nervous system (CNS) atrophy. Excess brain atrophy in MS has been interpreted as “accelerated aging.” Current paper tests an alternative hypothesis: MS causes CNS atrophy by mechanism(s) different from physiological aging. Thus, subtracting effects of physiological confounders on CNS structures would isolate MS-specific effects.

**Methods:**

Standardized brain MRI and neurological examination were acquired prospectively in 646 participants enrolled in ClinicalTrials.gov Identifier: NCT00794352 protocol. CNS volumes were measured retrospectively, by automated Lesion-TOADS algorithm and by Spinal Cord Toolbox, in a blinded fashion. Physiological confounders identified in 80 healthy volunteers were regressed out by stepwise multiple linear regression. MS specificity of confounder-adjusted MRI features was assessed in non-MS cohort (*n* = 158). MS patients were randomly split into training (*n* = 277) and validation (*n* = 131) cohorts. Gradient boosting machine (GBM) models were generated in MS training cohort from unadjusted and confounder-adjusted CNS volumes against four disability scales.

**Results:**

Confounder adjustment highlighted MS-specific progressive loss of CNS white matter. GBM model performance decreased substantially from training to cross-validation, to independent validation cohorts, but all models predicted cognitive and physical disability with low *p*-values and effect sizes that outperform published literature based on recent meta-analysis. Models built from confounder-adjusted MRI predictors outperformed models from unadjusted predictors in the validation cohort.

**Conclusion:**

GBM models from confounder-adjusted volumetric MRI features reflect MS-specific CNS injury, and due to stronger correlation with clinical outcomes compared to brain atrophy these models should be explored in future MS clinical trials.

## Introduction

Scientific advancements in Multiple Sclerosis (MS) translated into the development of disease-modifying treatments (DMTs) that inhibit central nervous system (CNS) tissue destruction if given to young subjects shortly after disease onset. Unfortunately, these treatments lose efficacy with advancing age of patients. On a group level, they show no statistically-significant inhibition of disability progression in subjects older than 54 years (1). To develop more effective MS treatments, we need sensitive outcomes that can quantify CNS tissue destruction against active comparator, in reasonably sized cohorts.

Magnetic resonance imaging (MRI) volumetric outcomes such as brain atrophy, have been successfully used in Phase II and Phase III MS clinical trials (2–4). However, is brain atrophy the most sensitive and the most specific imaging outcome in MS?

In terms of sensitivity, there is mounting evidence that atrophy of deep gray matter (GM) structures, especially thalamus (5), or enlargement of ventricles (6) exert larger effect sizes in MS than whole brain atrophy. Additionally, assembling several of these changing brain volumes into a single model using machine-learning (ML) algorithm(s) may further increase effect size.

In terms of specificity, brain atrophy also occurs during natural aging (7). In what we identified as the largest published study of healthy volunteers (HV; *n* = 2,790), physiological confounders such as age, gender and predicted intracranial volume (PIV), explained a very high proportion of variance in thalamic (>60%), caudate (>40%) and ventricular (57%) volumes (8). In fact, ML-derived models can predict chronological age [with a mean absolute error of +/-5 years (9)] using just brain MRI predictors. These are enormous effect sizes for physiological covariates and yet, most MS MRI studies do not subtract these confounder effects when analyzing correlations between MRI biomarkers and clinical disability (10).

In contrast, a study from European Magnetic Resonance Imaging in MS (MAGNIMS) consortium (11) examined the difference between chronological and brain MRI-predicted age, termed Brain-predicted Age Difference (Brain-PAD). The study demonstrated that 1204 MS patients had on average 6 years higher Brain-PAD compared to HV. Surprisingly, Brain-PAD measured in relapsing-remitting [RRMS] was not smaller than Brain-PAD in secondary- [SPMS] and primary-progressive MS [PPMS]. Brain-PAD also correlated with disability measured by Expanded Disability Status Scale (EDSS). Although no effect size was provided, the associated figures suggested that Brain-PAD explains <10% of EDSS variance. The authors concluded that MS is associated with accelerated brain aging (11).

However, there are important logical discrepancies with this interpretation: 1. If MS caused accelerated aging, then people with longer MS duration [i.e., progressive MS, (PMS)] should have higher Brain-PAD compared to people with shorter MS duration (i.e., RRMS). But this was not the case. 2. If MS causes accelerated brain aging and MS DMTs inhibit MS-associated CNS damage, then treated MS patients should have decreased Brain-PAD. But the authors saw the opposite: treated MS patients had significantly higher Brain-PAD compared to untreated MS patients (11). The alternative interpretation, consistent with the data, is that MS and natural aging destroy structurally overlapping CNS areas, but MS does so by different molecular mechanisms.

Indeed, physiological age can be also predicted with high accuracy by blood (12) or cerebrospinal fluid (CSF) biomarkers (13). Such CSF proteins-based molecular predictor of age failed to show accelerated aging in MS (13). These data strongly support the stated alternative interpretation, in which (molecular) mechanisms of aging and MS progression are mostly different.

The logical extension of this alternative interpretation is that aging and other physiological confounding effects on brain MRI volumes represent “noise” when measuring MS-specific processes. Here, we examine this hypothesis by addressing the following aims: (1) To adjust volumes of the CNS structures for physiological confounders to understand MS-specific effects on CNS structures; (2) To examine, whether confounder-adjusted MRI predictors can be assembled into models that predict clinical disability in the independent validation cohort, and whether such model(s) exerts larger effect size(s) than any single MRI biomarker; (3) To investigate whether computational model(s) derived from confounder-adjusted MRI predictors outperform models(s) from raw MRI volumes in predicting clinical disability outcomes.

## Materials and methods

**Figure 1:**
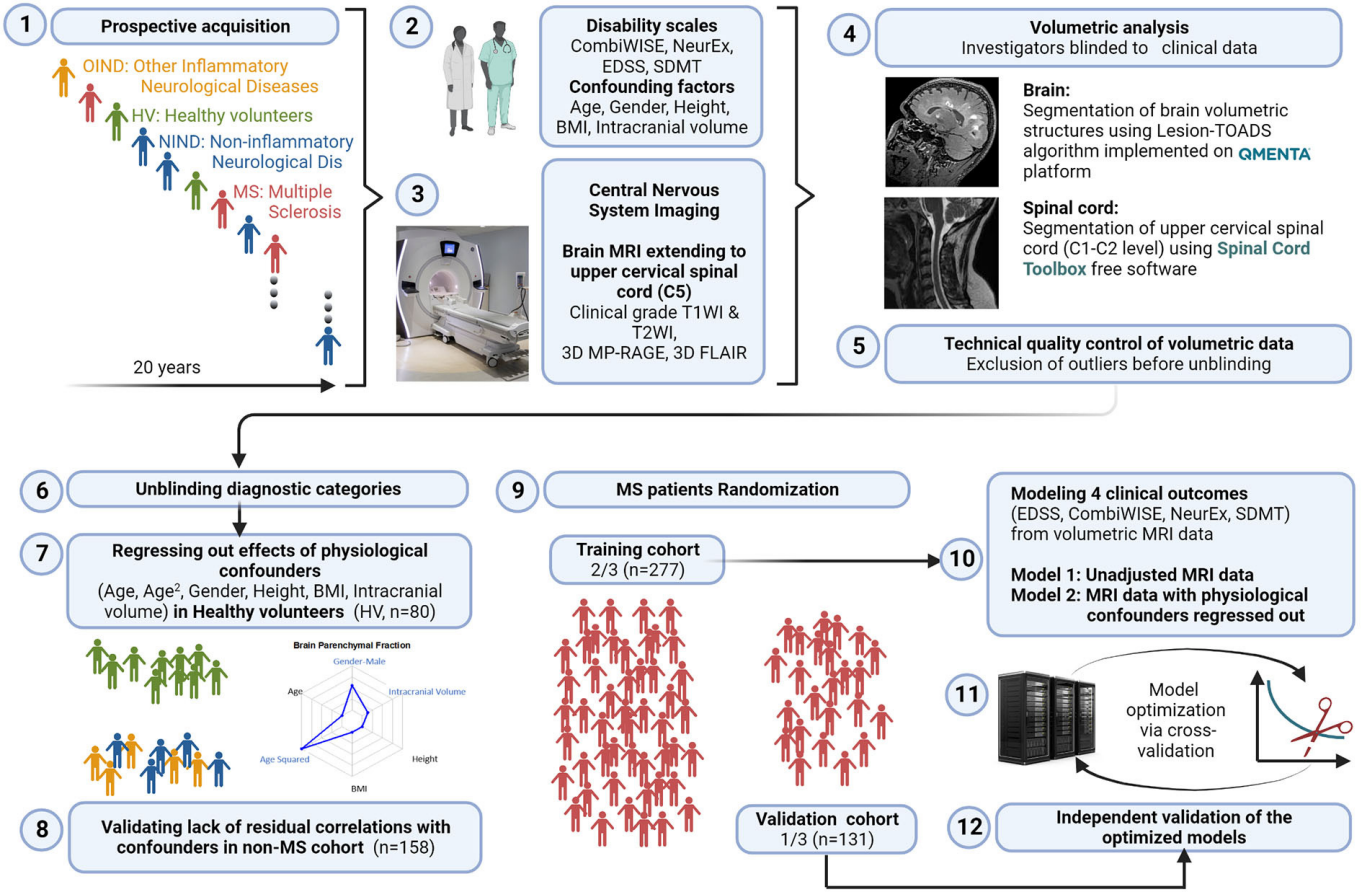
Study design. 1: All subjects participating in a prospective collection of standardized clinical and imaging outcomes under a natural history protocol for 20 years were enrolled. 2: All subjects underwent full neurological examination transcribed to the NeurEx™ App that automatically calculates clinician-derived disability scales. Additional functional tests, such as Symbol Digit Modalities Test (SDMT) and stated confounding factors were collected and transcribed to research database. 3: All subjects underwent research brain MRI that extended caudally to the C5 level of the spinal cord (SC). 4: Anonymized MRIs were uploaded to the cloud-based QMENTA platform to derive brain volumetric data using Lesion-TOADS algorithm. Upper cervical SC C1-C2 volume was calculated using Spinal Cord Toolbox. 5: Resulting quantitative MRI biomarkers were assessed for quality to identify intra- and inter-individual outliers. Identified outliers were manually checked and scans with incorrect segmentation were excluded (122/768 = 15.9%). 6: Unblinding of diagnostic categories occurred after exclusion of technical outliers was completed. 7: We regressed out the effects of six stated confounders measured in the HV cohort (*n* = 80) and applied the same transformation to non-MS (*n* = 158) and MS (*n* = 408) cohorts to eliminate effects of physiological confounders on MRI volumes. 8: We validated lack of residual correlations with the confounding factors in non-MS cohort. 9: MS patients were randomly split into training (*n* = 277) and validation (*n* = 131) cohorts using stratified split to assure equal proportions of gender and MS types in both cohorts. 10: Gradient boosting machine (GBM) algorithm was applied to the training cohort using confounder-adjusted (and unadjusted) MRI features as predictors to derive two sets of models for the four stated clinical outcomes. 11: Models were further optimized in the training cohort using 10-fold cross validation. 12: Resulting eight models were applied to the independent validation cohort that did not contribute, in any way, to the generation or optimization of the models. Created with BioRender.com.

The study design is shown in Figure 1.

### Cohort characteristics

All subjects (MS and non-MS patients and HV) were prospectively recruited into the protocol “Comprehensive Multimodal Analysis of Neuroimmunological Diseases of the Central Nervous System” (Clinicaltrials.gov Identifier: NCT00794352) and provided written informed consent. The inclusion criteria for patient cohort are age at least 12 years and clinical symptoms, CSF results or MRI imaging suggestive of neuroimmunological disease. Approximately 60% of enrolled patients eventually fulfill contemporary version of MS diagnostic criteria (with all 3 MS subtypes represented), while 20% have other inflammatory neurological diseases (OIND) and 20% have non-inflammatory neurological diseases (NIND). The HV inclusion criteria are age at least 18 years, absence of known diseases and conditions that could affect CNS and normal vital signs at the screening visit. The HV cohort undergoes exactly same procedures (including same MRI) as patients. The protocol was approved by the Combined Neuroscience Institutional Review Board of the National Institutes of Health. Patient demographics and other clinical characteristics are provided in Supplementary Figure 1. Note that despite lower age limit of inclusion criteria, the youngest MS patient was 18 years old.

All subjects (*n* = 646) with brain MRI images that passed quality control (see below) and had matched clinical outcomes were included. Neurological exams documented in structured electronic medical record note were either transcribed (before 2017) or directly documented (after 2017) into the NeurEx^TM^ App (14) by clinicians with MS specialization. The NeurEx^TM^ App automatically computes MS disability scales including EDSS (15) (ordinal scale from 0 to 10) and NeurEx (continuous scale from 0 to theoretical maximum of 1349).

Functional tests (i.e., timed 25 foot walk and 9 hole peg test) are required for computing of Combinatorial weight-adjusted disability score (CombiWISE; continuous scale from 0 to 100) (16), and Symbol Digit Modalities Test (SDMT) (17). These were acquired by investigators blinded to clinician-derived disability scales and were recorded into research database.

### Volumetric analysis

Investigators involved in MRI analysis were blinded to diagnostic categories and clinical outcomes. MRIs were performed on two scanners: Signa (3TA, General Electric, Milwaukee, WI) using 16-channel head coil and Skyra (3TD, Siemens, Malvern, PA) using 32-channel head coil. Sequences included 3D-MPRAGE (TR, 3000 ms; TE, 3 ms; TI, 900 ms; FA 8°; 1-mm isotropic resolution, TA 6 min), 3D-FLAIR (TR, 4800 ms; TE, 354 ms; TI, 1800 ms; 1-mm isotropic resolution; acquisition time, 7 min), and PD/T2 (TR, 3540 ms; TE, 13 and 90 ms; 0.8-mm in-plane resolution; slice thickness, 2 mm; acquisition time, 4 min) on 3TD and 3D-FSPGR-BRAVO (TR, 1760 ms; TE, 3 ms; TI, 450 ms; FA 13°; 1-mm isotropic resolution; acquisition time, 5 min), 3D-FLAIR-CUBE (TR, 6000 ms; TE, 154 ms; TI, 1800 ms; 1-mm isotropic resolution; acquisition time, 9 min), and PD/T2 (TR, 5325 ms; TE, 20 and 120 ms; 1-mm in-plane resolution; slice thickness, 3 mm; acquisition time 4 min) on 3TA. Sagittal and axial cuts extended distally to C5 level, allowing determination of medulla/upper spinal cord (SC) atrophy using SC Toolbox (18).

After pre-processing steps (1. De-identification, 2. DICOM to NIFTI transformation, 3. Skull stripping; and 4. Alignment), images were uploaded to commercial cloud-based imaging platform QMENTA (https://www.qmenta.com/) which, in collaboration, implemented the published Lesion-TOADS (19) algorithm. Lesion-TOADS combines a topological and statistical likelihood atlas for computation of 12 CNS volumetric biomarkers: Cerebral white matter (WM), Cerebellar WM, Brainstem, Putamen, Thalamus, Caudate, Cortical gray matter (GM), Cerebellar GM, Lesion Volume, Ventricular CSF and Sulcal CSF. Average cross-sectional area (CSA) of the upper cervical SC at C1-C2 level was calculated using SC Toolbox from 3D MPRAGE brain MRI images.

Before unblinding, we performed quality control of volumetric data. A total of 122 (15.9%) MRI scans was excluded due to inaccurate segmentation of brain structures, low image quality, motion artifacts, or as intraindividual outliers leaving 646 scans for the final dataset.

After unblinding diagnostic categories, MS patients were randomly split into training and validation cohort (2:1 ratio) with equal proportion in gender and MS subtypes.

### Adjusting MRI biomarkers for healthy volunteers confounders

**Figure 2:**
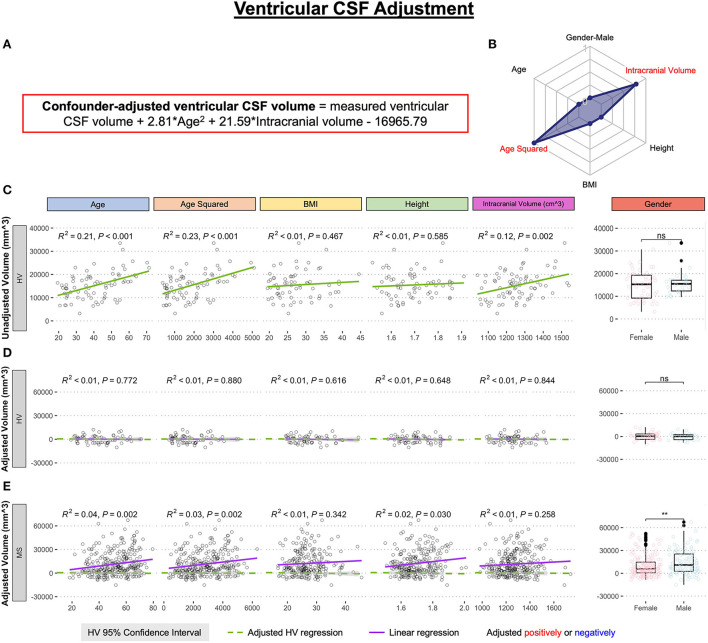
Example of the adjustment of single MRI biomarker (i.e., ventricular CSF volume) for six physiological confounders. **(A)** The final equation for the confounder-adjusted ventricular volume is shown at the top of the Figure in red outline. This equation was derived from multiple linear regression models as described in the Method section. **(B)** In the top right corner is resulting radar chart that shows proportional weights of the applied confounder adjustment, with confounders with lowest weights (i.e., the innermost circle) representing zeros. **(C)** Univariate linear regression models between each tested confounder on x-axis (first 5 graphs) or gender (sixth graph) and measured ventricular volume on the y-axis in 80 healthy volunteers (HV). **(D)** Same univariate regressions in the HV cohort after applying adjustment formula show no remaining effect of confounders. **(E)** Applying HV-derived adjustment formula to MS cohort shows remaining significant residual effect of age, when considering Bonferroni adjustment for multiple comparisons (i.e., *p* < 0.05/12 = *p* < 0.004). Analogous Figures showing adjustment for all MRI features are in the Supplementary material. ns, not significant, **P < 0.01.

Using only the HV cohort (*n* = 80), MRI volumes were adjusted for physiological confounders that were previously shown to influence brain MRI volumes in the HV (8): age, age^2^ (to capture non-linear rates of atrophy), body mass index (BMI), height, gender, and supratentorial intracranial volume; using stepwise multiple linear regression. Final linear regression models (equations provided in the appropriate figures) were applied to all subjects to regress out physiological confounders. The example of confounder adjustments for ventricular volume is shown in Figure 2. Supplementary material contains analogous data for all remaining MRI biomarkers.

The correlation between the confounders and individual structural volumes, before and after covariate adjustments, was evaluated using linear regression models in R Studio, reporting a coefficient of determination (R^2^; the proportion of variance explained ranging from 0 to 1, where higher number signifies closer fit of experimental data points to the linear regression line of the model) and HV 95% confidence interval.

Differences between HV and MS cohorts in unadjusted and confounder-adjusted MRI biomarkers were analyzed using unpaired two-samples Wilcoxon signed-rank test in R (20).

### Gradient boosting machine modeling in the MS training cohort

Unadjusted or confounder-adjusted MRI biomarkers that showed statistically significant difference between MS and HV in univariate analyses were used as predictors to model four clinical outcomes: CombiWISE, EDSS, NeurEx, and SDMT. We selected a tree-based supervised ML algorithm hypothesizing that MRI features may have non-linear effects and patients may exert heterogeneity in which brain/SC structures are affected by the disease. Among tree-based algorithms, we selected GBM; while GBM is more difficult to optimize, it is believed to generally outperform Random Forest. GBM builds trees sequentially, where each successive tree is built using residuals from the previous tree's predictions. The predictions are iteratively updated by adding the current tree's prediction (times a shrinkage parameter) to the previous tree's prediction. For each tree constructed, an out-of-bag (OOB) sample containing half of the observations is withheld from the training cohort to introduce randomness into the modeling process. Main GBM tuning parameters are the depth of the individual trees (interaction depth), the shrinkage parameter (learning rate), the minimum number of observations in trees' terminal nodes, and the number of trees. Using the *gbm* R package (21), we selected an interaction depth of 6, nodes of 5, a shrinkage parameter of 0.01, and used a 10-fold cross validation to select the optimal number of trees that will prevent each model from overfitting. Improvement in mean squared error from splits within each individual tree and the average of these improvements across all trees in the ensemble calculated the relative influence of each variable in a model.

Each clinical scale was modeled separately since each assesses different neurological functions (e.g., SDMT measures reaction time reflective of cognitive disability whereas other three scales reflect predominantly physical disabilities). The models were optimized by observing the lowest root mean squared error in the combination of feature selections within the MS training cohort.

### GBM model validation

With the final optimized models, two validations methods were performed: (1) 10-fold cross-validation and (2) an independent cohort validation. Ten-fold cross-validation reuses the training cohort data by randomly partitioning the data into an “internal” training set (90% of the total training cohort) and validation set (10% of the total training cohort) on different iterations. The model then tests prediction accuracy of the withheld samples.

The independent cohort, a gold-standard validation technique, utilizes a completely new dataset that did not participate in the model development or optimization in any way.

Correlations between measured and predicted clinical scores were evaluated using linear regression models in R Studio version 4.1.0 (22, 23), reporting R^2^, Spearman Rho (the relationship strength of the predicted and measured scores) from R CRAN Package: stats (20) and rstatix (24); and Concordance Correlation Coefficient (CCC; the degree of reliability in the method when comparing two measurements of the same variable) from R CRAN Package: DescTools (25).

## Results

### Regressing out physiological confounders

We regressed out effects of physiological confounders based on internal HV cohort as described in Methods and exemplified in Figure 2. For ventricular CSF volume, the multiple linear regression model selected only effects of age^2^ and intracranial volume from the 6 tested confounders. For some other CNS structures, the multiple linear regression models were more complex (Supplementary material), but consistent with published studies examining effects of covariates on CNS volumes in much larger HV cohorts (8).

### MS-specific residual effects of age and gender on CNS volumes

**Figure 3:**
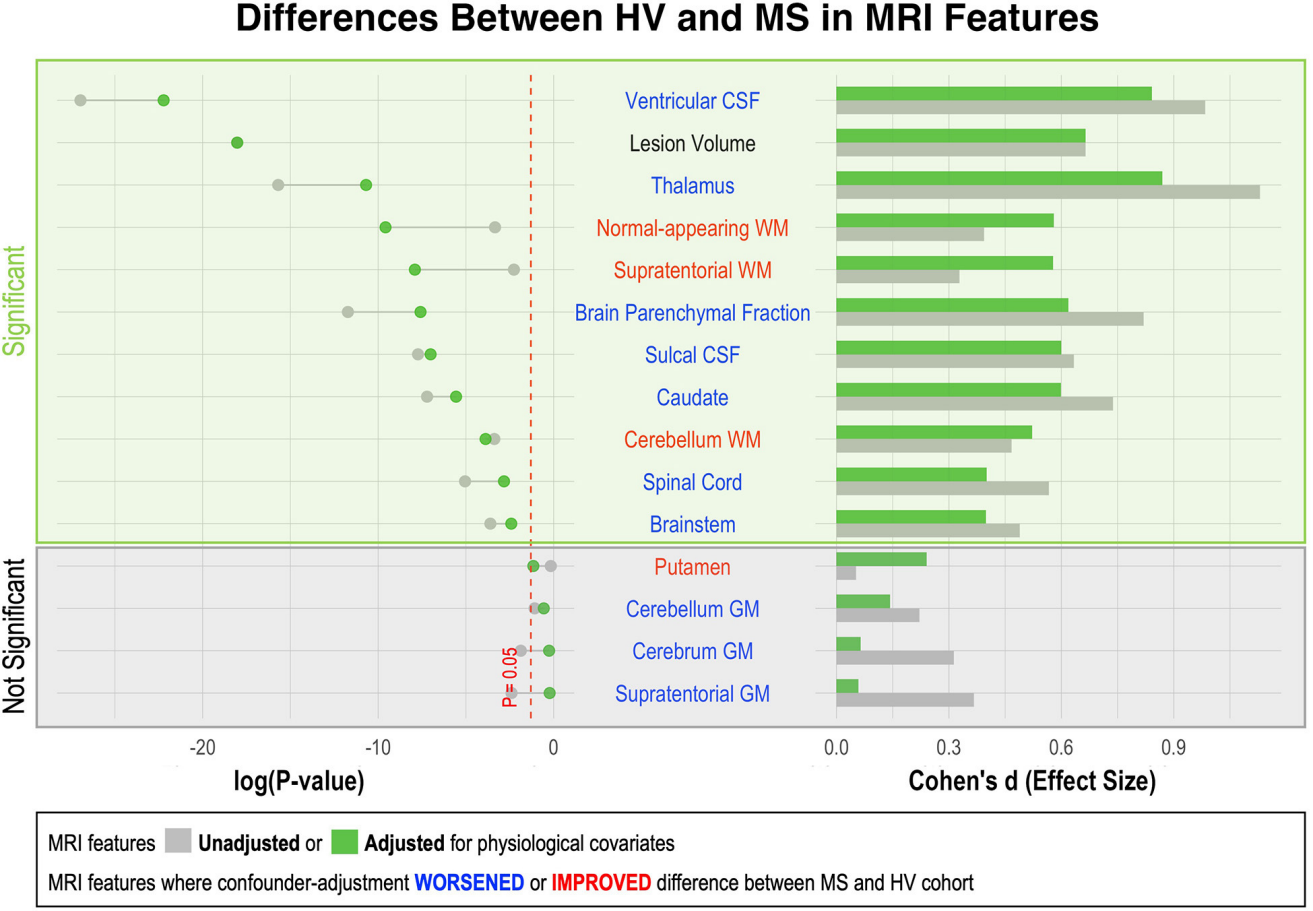
Effect of adjustment for physiological confounders on measured differences in MRI volumetric features between HV and MS patients. Right side of the figure shows effect sizes of unadjusted (gray bars) and confounder-adjusted (green bars) volumetric MRI features to differentiate MS from HV. Effect sizes are shown as Standardized Mean Difference (i.e., Cohen's d). Left side of the figure shows the effect of applied confounder adjustment with log *p*-value. MRI features where confounder adjustment decreased the difference between MS and HV are highlighted in blue, whereas those MRI features where confounder adjustment increased the difference are highlighted in red. Eleven out of 15 MRI features differentiated MS from HV with statistical significance. The difference between MS and HV was partially driven by confounder differences in majority of these MRI features (6/10). Thus, the applied adjustment decreased the ability of these features to differentiate MS from HV. On the other hand, three measurements of white matter volume (supratentorial WM, normal-appearing WM and cerebellum WM) enhanced their ability to differentiate MS from HV after confounder adjustment.

**Figure 4:**
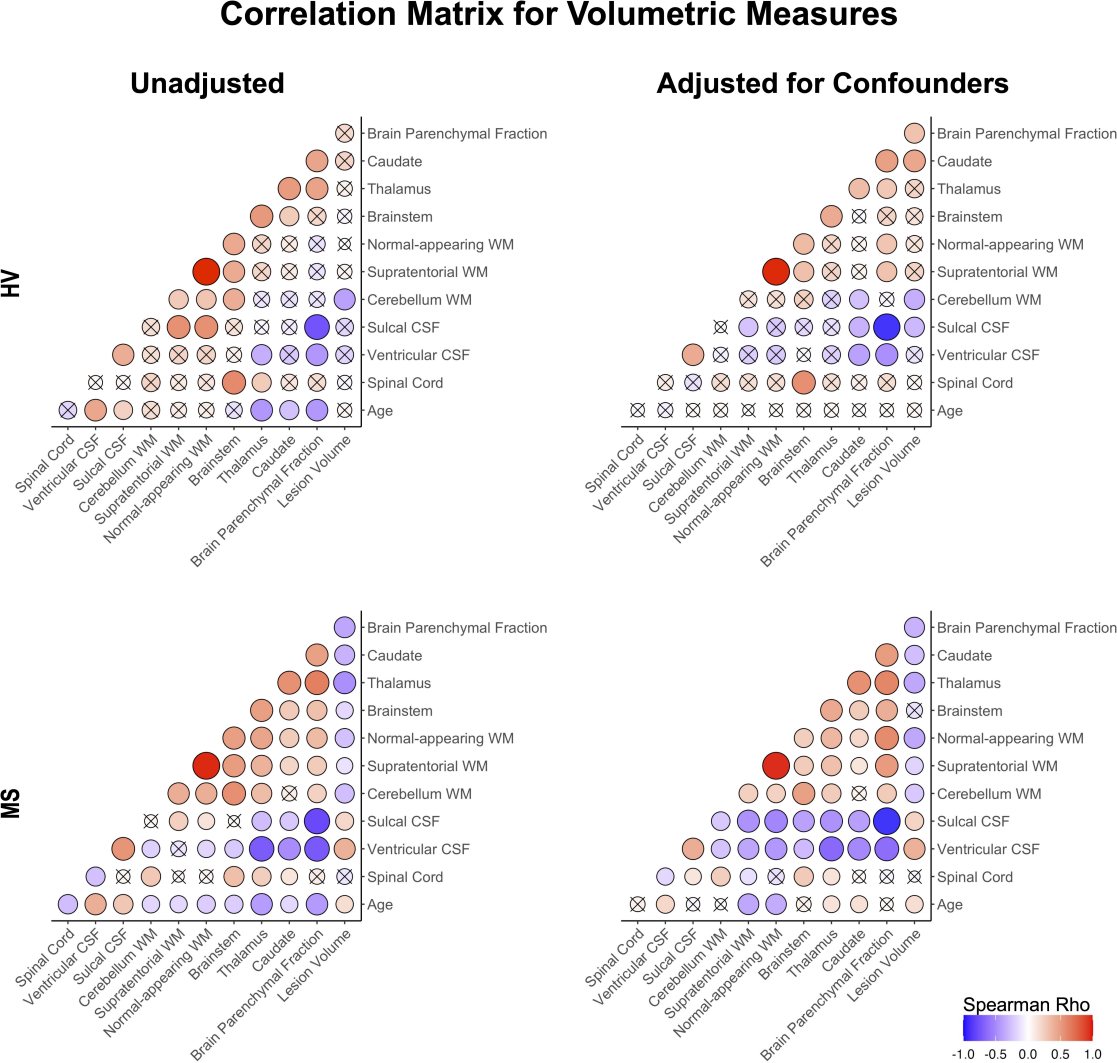
Correlation matrix of unadjusted and confounder-adjusted MRI features. The top row shows correlations in HV cohort. Bottom row shows correlations in MS training cohort. Left panel shows correlations of unadjusted MRI features. Right panel shows correlations of confounder-adjusted MRI features. Correlations that are not statistically significant are marked as (x), positive correlations are marked in red and negative in blue colors. The size of the circle corresponds to Spearman's correlation coefficient. Confounder adjustment eliminated correlations of MRI features with age, while it generally strengthened correlations of brain parenchymal fraction with remaining MRI features.

Consistent with published literature, we observed decrease in all unadjusted GM volumes with age in MS, HV and non-MS subjects (Supplementary Figure 2) and significant decrease in most GM unadjusted volumes in MS vs. HV cohorts (Figures 3, 4). However, we observed that the difference between MS and HV for all GM volumes was highest in young (RRMS) patients compared to older (PMS) patients (Supplementary Figure 2). Consequently, when MRI volumes were adjusted for confounding factors identified in HV cohort, we observed paradoxical positive correlation of confounder-adjusted GM volumes with age in MS cohort, that reached statistical significance (i.e., Bonferroni adjusted *p* < 0.004) for caudate (*R*^2^ = 0.04, *p* = 0.002; Supplementary Figure 7), cerebrum GM (*R*^2^ = 0.06, *p* < 0.001, Supplementary Figure 10), putamen (*R*^2^ = 0.08, *p* < 0.001; Supplementary Figure 12) and supratentorial GM (*R*^2^ = 0.06, *p* < 0.001, Supplementary Figure 15). Since these residual effects were not seen in non-MS cohort (Supplementary Figure 2), they are MS-specific and are driven by non-physiologically low GM volumes in young patients.

In contrast, we observed residual loss of supratentorial WM (*R*^2^ = 0.10; *p* < 0.001) and normal appearing WM (*R*^2^ = 0.09; *p* < 0.001; Supplementary Figure 3) with MS aging, paralleled by significant residual increase in ventricular (but not sulcal) CSF volume (*R*^2^ = 0.04; *p* = 0.002). This progressive non-physiological WM loss also affected the cerebellum WM, even though the residual correlation of cerebellum WM with age in MS became non-significant after Bonferroni adjustment. This loss of WM was MS-specific: the large non-MS cohort (*n* = 158) behaved identically to HV regarding all WM volumes. In contrast, non-MS cohort exhibited non-physiological atrophy in some GM structures (including thalamus, Supplementary Figure 4A) and non-physiological increase in ventricular volume (Supplementary Figure 4B).

We conclude that subtracting effects of physiological confounders on volumes of CNS structures identified non-physiological loss of GM volumes in young (RRMS) MS patients and MS specific, progressive loss of WM volumes across MS lifespan.

Additionally, subtracting physiological covariates highlighted gender effects in MS, with males having significantly higher lesion load and ventricular volume compared to females (Figure 2E, Supplementary Figure 18E).

### Differences in unadjusted and confounder-adjusted MRI features between HV and MS

As shown in Supplementary Figure 1, HV cohort was significantly younger than MS (i.e., Median 40.4 years vs. Media*n* = 52.4 years; *p* = 1.24e-7). Because most CNS volumes decrease with age, adjusting for covariates diminished differences between HV and MS cohorts in most CNS volumetric biomarkers (Figure 3). The greatest decrease in effect size was seen for thalamus, caudate and brain parenchymal fraction (BPFr) - consistent with the reported strong effect of physiological aging on these CNS structures (8).

Conversely, we observed increase in effect sizes of WM volumes on differentiating MS from HV after confounder adjustment: supratentorial WM and normal appearing WM, but marginally also cerebellum WM.

Confounder adjustment affected correlations between MRI features (Figure 4). As expected, it eliminated correlations of MRI biomarkers with age in the HV cohort. But the adjustment also enhanced correlations of BPFr with most brain volumes in both HV and MS cohorts. In MS, confounder adjustments strengthened correlations of brain WM volumes and ventricular CSF with remaining MRI features.

Strengthening correlations between MRI features after confounder adjustment indicate that different physiological confounders affect different CNS structures (Supplementary material) (8). This supports the hypothesis that confounder effects represent noise when analyzing effects of MS on CNS structures.

### Univariate correlations of MRI features with clinical outcomes

**Figure 5:**
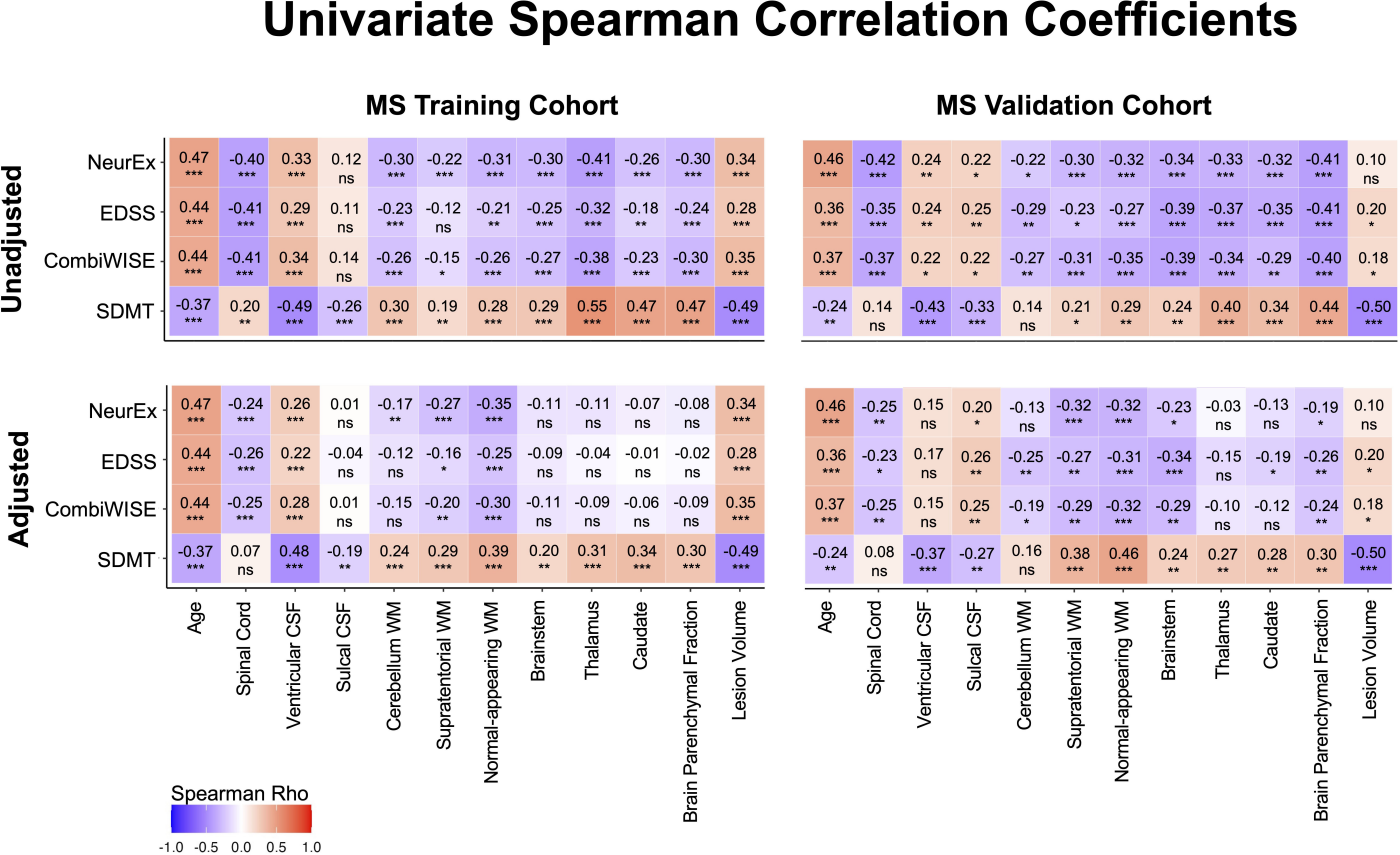
Unilateral correlation matrix of unadjusted and confounder-adjusted MRI features with disability outcomes. The top row shows unilateral correlations of disability outcomes with unadjusted MRI features. Left panel shows correlations of MS training cohort and right panel shows correlations of MS validation cohort. Bottom row shows analogous correlations with confounder-adjusted MRI features. Correlations that are not statistically significant are marked as (ns). Positive correlations are marked in red and negative in blue colors. Correlation *p*-values indicate statistical significance (*P < 0.05, **P < 0.01, and ***P < 0.001). Additional descriptive statistics are shown in Supplementary Table 1.

Spearman correlation coefficients (and *p*-values) of unadjusted MRI volumes with clinical outcomes (Figure 5, Supplementary Table 1) show that age correlates stronger with physical disability outcomes (EDSS, CombiWISE and NeurEx) than any MRI biomarker. This observation, underappreciated in MS field, not only highlights the dominant effect of age on physical disability outcomes but also raises a question whether (rather weak) correlations of brain volumes with MS disability outcomes are primarily driven by their (mutual) correlations with age. In contrast, lesion volume and ventricular CSF outperformed age in correlating with SDMT, an outcome focusing on cognitive disability. These correlations were completely reproduced in the independent validation cohort of MS patients (Figure 5, right panels).

If the shared effect of age on MRI volumes and disability outcomes explained all variance, then subtracting effect of physiological confounders on MRI volumes should eliminate correlations of all (adjusted) CNS volumes with MS clinical outcomes. But this is not what we observed: confounder-adjusted spinal cord volume, supratentorial and normal appearing WM volumes and lesion load remained reproducibly significantly associated with EDSS, NeurEx and CombiWISE. For SDMT, spinal cord volume did not correlate, and other than cerebellum WM, all remaining covariate adjusted MRI volumes remained significantly and reproducibly correlated with cognitive disability.

We conclude that brain volumes are stronger determinant of cognitive disability in MS than age, while age has stronger correlation with physical disability outcomes than any CNS volume. However, even after subtracting effects of physiological aging (and other confounders) on MRI volumes, spinal cord volume, lesion load and supratentorial WM volumes continue to correlate with physical disability outcomes, demonstrating effect of MS on these CNS structures beyond natural aging.

### Models from MRI volumetric data adjusted for physiological confounders achieve stronger effect sizes in predicting MS clinical outcomes in the independent validation cohort

Unadjusted and confounder-adjusted MRI features that reproducibly differentiated HV and MS cohorts were inputted into ML-based models of four clinical outcomes. Because age and gender exerted significant influence on clinical outcomes and their effects were subtracted from confounder-adjusted MRI volumes, we added these demographic variables into the model that used adjusted MRI volumes. The full statistical results from all models are in Supplementary Table 2.

**Figure 6:**
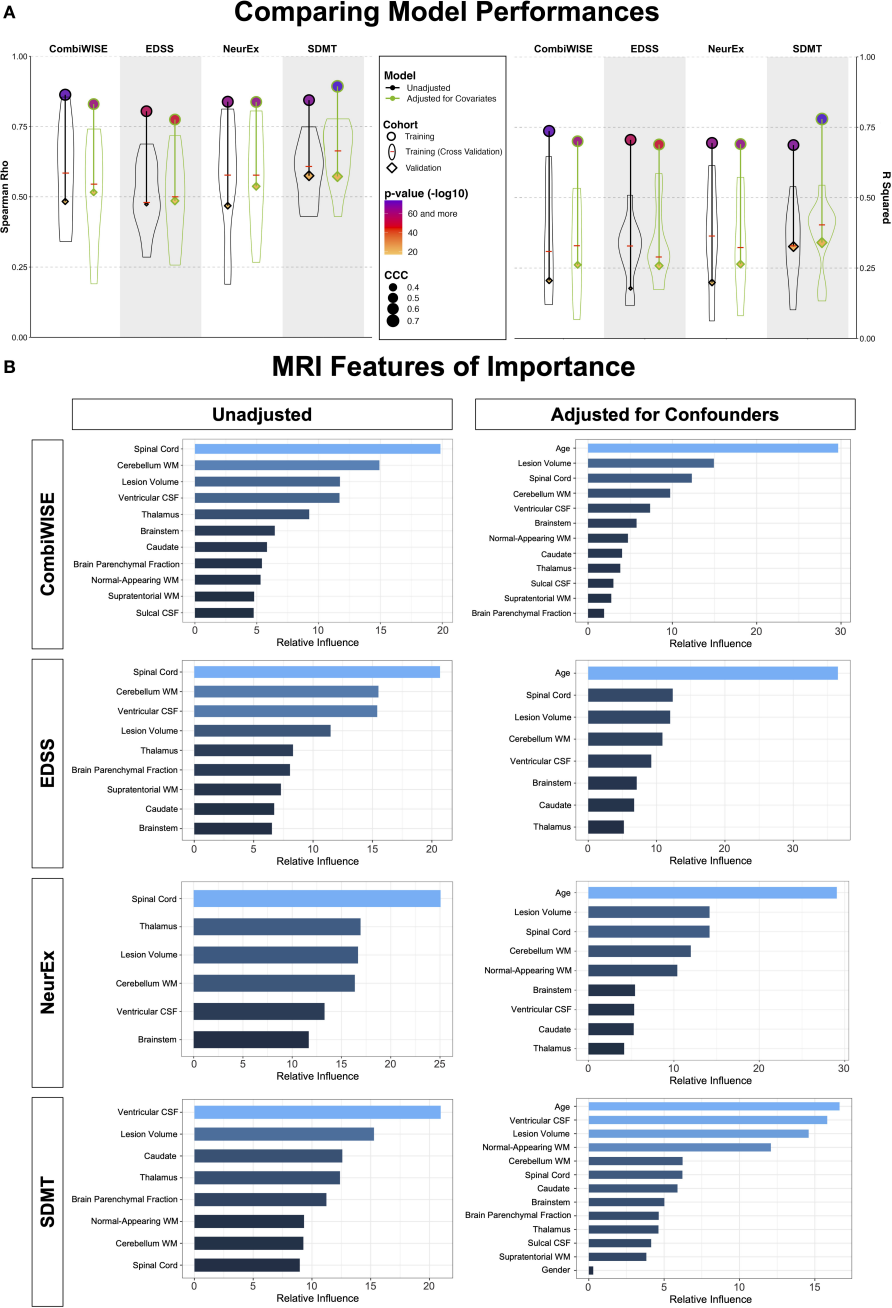
Summary of model performances. **(A)** The left panel shows Spearman Rho correlation coefficients while right panel shows coefficient of determination (*R*^2^) for the same 8 models. For each outcome (arranged in vertical columns), the unadjusted models are on the left side outlined in black, and the confounder-adjusted models are on the right side, outlined in green. The model performance in the training cohort is shown as circle; the distribution of cross-validation (i.e., re-using of the training cohort) results is shown as a violin plot with the median marked as a red horizontal line; the independent validation cohort performance is shown as diamond. The size of the symbols (i.e., circle and diamond) correspond to the Linh's concordance correlation coefficient (CCC). Finally, the color of each symbol represents *p*-value (-log10) with lower *p*-values displayed in purple color and higher *p*-values in orange in accordance with the heatmap displayed in the figure legend. Unequivocal and robust decrease in model's performance is seen from training cohort to cross-validation to independent validation. Detailed statistics of the model performances are shown in Supplementary Table 2. **(B)** For each clinical outcome modeled (presented in rows) we show number of features selected in the final model(s) arranged in descending order of variable importance. The unadjusted models are displayed on the left, while confounder-adjusted models are displayed on the right.

In the training cohort (represented by circles in Figure 6A), models from raw MRI biomarkers (circles with black outlines) exerted stronger effect sizes compared to models from covariate-adjusted MRI biomarkers (circles with green outlines) for physical disability outcomes (i.e., CombiWISE, EDSS, NeurEx). Model from covariate-adjusted MRI biomarkers outperformed model from raw MRI biomarkers for SDMT.

However, ML-algorithms are extremely powerful: they can use spurious observations (i.e., noise) to derive overly optimistic models. Therefore, ML-derived models must be validated to derive better estimate of their true effect sizes, even though this is done in only 15% of MS publications based on recent meta-analysis (10).

There are two approaches to model validation: one reuses the training cohort data and is generally called “cross-validation.” We used 10-fold cross-validation, shown in Figure 6A as violin plots with medians marked by red cross-sectional line. Cross-validation have broad distribution of effect sizes in comparison to the full training cohort, showcasing the poor estimate of model's performance depending on the training cohort splits. Nevertheless, cross-validation medians had uniformly lower effect sizes than the training cohort; the decrease in effect sizes was substantial (between 40 and 60% for *R*^2^; Supplementary Table 2).

However, because all training cohort data contributed to some aspects of model development (e.g., selection of MRI outcomes for modeling), the gold standard of assessing model performance is applying the final model to the independent validation cohort. This type of validation is performed in <8% of published MS studies (10).

The independent validation cohort (shown as diamonds in Figure 6A) achieved effect sizes consistently below the cross-validation medians, showcasing that cross-validation medians still over-estimate models' performance. Nevertheless, all eight models validated with very low *p*-values (all below 9e-8; Supplementary Table 2).

Most importantly, confounder-adjusted models consistently outperformed models from unadjusted features in the independent validation cohort. The absolute difference in *R*^2^ values between unadjusted and adjusted models was up to *R*^2^ = 0.08 (i.e., EDSS unadjusted model achieved *R*^2^ = 0.18, while adjusted model had *R*^2^ = 0.26; Supplementary Table 2). This represents relative improvement of 30%.

We conclude that the stronger validation performance of confounder-adjusted models supports our hypothesis that effects of aging and other physiological covariates on MRI volumes represents noise when predicting MS-related clinical disability.

This does not indicate that age does not play role in MS. In fact, age was selected as the strongest feature in all confounder-adjusted models (Figure 6B), implying that age is the most important determinant of MS-related disability. Our results simply support the hypothesis that age exerts effects on CNS structures by (mostly) different mechanisms in physiological aging and in MS. By separating the effect of age on MS from its effect on brain structures during natural aging, we built more reliable models that predict MS-associated disability with higher effect sizes in the independent validation cohort.

### Validated models from covariate-adjusted MRI biomarkers predict MS physical (EDSS) and cognitive (SDMT) disability with comparably highest effect sizes in reported literature

Thanks to recent meta-analysis of 302 papers describing models of MS clinical outcomes (10), we can compare our results with other published MRI biomarker-based models. Using an associated website that allow users to dynamically explore this rich dataset, we identified 40 papers that used MRI biomarkers to model EDSS as ordinal scale and reported *p*-values, and 20 papers that reported effect sizes as *R*^2^.

Studies that use small cohorts or fail to implement methodological design limiting bias can overestimate effect sizes (26–28). Thus, the meta-analysis scored methodological rigor of reviewed studies by grading 7 criteria: 1. Blinding, 2. Defined strategy to deal with outliers, 3. Explanation of missingness, 4. Adjustment for confounders, 5. Number of comparisons made and whether *p*-values were adjusted, 6. Presence of controls and 7. Validation (cross-validation of the training cohort vs. independent validation cohort).

The median numbers of criteria fulfilled by published studies that modeled EDSS was 2, and the majority studied <100 MS patients. Only 25% of studies applied covariate adjustments. Only 38% of studies adjusted *p*-values for multiple comparisons, and studies that did not adjust performed up to 500 comparisons. The studies with the highest methodological rigor and largest cohorts originated from the MAGNIMS consortium. MAGNIMS study of effects of GM brain volumes on differentiating MS (*n* = 961) from HV (*n* = 203) and on disability prediction found negative association between deep GM (β = −0.71; *p* < 0.0001) and cortical GM (β = −0.22; *p* < 0.0001) and EDSS. Unfortunately, *R*^2^ was not reported. For much smaller studies that reported *R*^2^, the range was 0.7 to 0.05 in the training cohort. Only one study reported cross-validation results (29) and achieved *R*^2^ = 0.19 (*p*-value range from <0.001 to 0.04) in RRMS (*n* = 250) and *R*^2^ = 0.16 (*p*-value range from 0.02 to 0.04) in PMS (*n* = 114).

Current study fulfills 7/7 criteria of methodological rigor and is the only study that includes independent validation cohort. For the confounder-adjusted EDSS model, the training cohort *R*^2^ is 0.69 (*p* = 3.8e-43). Median cross-validation *R*^2^ is 0.29 and the independent validation cohort *R*^2^ is 0.26 (*p* = 2.4e-08). To our knowledge, this is the strongest reported effect size for predicting EDSS as ordinal scale from quantitative MRI biomarkers in the literature thus far.

Analogously, we identified 12 studies that used MRI predictors for modeling SDMT. 11/12 reported *p*-values and 5/12 reported *R*^2^. The median methodological rigor score was 2/7 and most cohorts were smaller than 100 subjects (the largest had 151 subjects). Again, only 25% applied covariate adjustments. Only 16.7% adjusted for multiple comparisons and the studies that did not adjust *p*-values performed up to 100 comparisons. No studies reported cross-validation or independent validation. The reported R^2^ (for the training cohorts) ranged from 0.62 to 0.3. Again, our confounder-adjusted SDMT model achieved *R*^2^ = 0.78 (*p* = 7.3e-75) with independent validation *R*^2^ = 0.34 (*p* = 2.9e-11), which represents the best performance among published studies.

## Discussion

The goal of this project was to gain understanding of MS-driven volumetric MRI changes and to examine if computational models of confounder-adjusted MRI volumes reproducibly predict different clinical outcomes.

Before we discuss our findings, we want to point out following limitations: our HV cohort is relatively small and the Lesion-TOADS automated segmentation algorithm, implemented from the inception of our natural history protocol, has relatively narrow adoption. Despite the differences in cohort sizes and the segmentation algorithms, our observations are aligned with published literature, both for HV (8) and MS (30, 31). Specifically, although we tested six confounders, only age (and age^2^), gender and total intracranial volume influenced volumetric brain MRI features with effect sizes comparable to those reported previously (8). Second, we did not examine the interaction between confounders because others (8) found that these had negligible effects. To facilitate broader use and potential external validation of our results, we collaborated with QMENTA to implement publicly available Lesion-TOADS algorithm on their platform. The other important limitation is that by the virtue of being national referral center, our patient population may be skewed toward subject with more aggressive disease. However, this limitation applies to all MS imaging studies, as these are performed almost uniformly in tertiary academic centers.

Notwithstanding these limitations, our study achieved its aims and highlighted under-appreciated aspect of age (and other physiological covariates) on CNS volumes that are generally believed to reflect solely MS disease process. Instead, age had consistently stronger univariate correlations with MS physical disability than any single MRI biomarker. In other words, age of the MS patient more accurately predicts his/her physical disability than any single volumetric CNS biomarker, including upper cervical spinal cord volume. Ignoring such large effects of confounders on MRI volumes overestimates how well the measured change reflects MS progression. It also poses problem for development of new treatments, especially for progressive MS patients who no longer form new lesions, and thus Phase II trials can't use contrast-enhancing lesions (CELs) as outcome. If physiological aging exerts stronger effect size on brain volumes than MS progression, then treatments that target MS-specific process may have limited effect on brain volumes (e.g., brain atrophy), which is precisely what was seen in the trial of lamotrigine in SPMS (32).

These observations support underlying premise for this work, that if we want to understand disease-specific effects and test disease-targeting treatments, we cannot ignore physiological confounders with large effect sizes. Indeed, correcting for covariates provided two important insights in MS-specific effects on brain tissue: very early decrease in GM volumes in young MS patients and progressive, non-physiological loss of CNS WM volumes.

How do we explain the apparent discrepancy between MS-specific effects on GM vs. WM volumes when these are biologically related (i.e., axonal loss must lead to neuronal loss)? Unfortunately, processes such as edema, inflammation, gliosis and restructuring of extracellular matrix may mask cellular (e.g., neuronal) loss during disease process. Even relative stability of T2 lesion load after patients stopped forming new lesions may be molecularly dynamic, where spread of diseased tissue at lesion edge (e.g., in chronic active lesions) maybe compensated by the volume collapse at severely affected lesion center. Because volumetric MRI can't differentiate such molecularly distinct processes, all we can conclude that after subtracting the effects of natural aging and sexual dimorphism, WM pathology (represented by formation of MS lesions associated with atrophy of deep GM structures and enlargement of ventricles) is the dominant effect of MS visible on conventional volumetric brain MRI. The resulting correlation matrix of confounder-adjusted MRI volumes supports this conclusion by demonstrating relatedness of MS-induced changes in all aforementioned structures. Similarly, these related brain structures were selected by most GBM models, with predictably higher influence of WM volumes in the models based on confounder-adjusted biomarkers.

Our GBM models from confounder-adjusted MRI features predicted four MS disability scales with high statistical significance in the independent validation cohort. These models outperformed analogous models derived from unadjusted MRI features and significantly outperformed any single MRI biomarker in the independent validation cohort.

Our results also highlight the limitation of studies that model MS clinical outcomes in the single cohort and do not validate model performance in the new set of patients who did not contribute to model generation or optimization. We show that training cohort data, including cross-validation, overestimate effect sizes and do not reliably predict the performance of the models in the independent validation cohort. Specifically, apparently stronger models derived from raw (covariate unadjusted) MRI biomarkers in the training cohort under-performed compared to covariate-adjusted models in the independent validation cohort, for all outcomes tested.

This is consistent with our extensive observations using independent validation in all our projects (33–36). Therefore, while cross-validation should be included in all modeling studies, the independent validation must be considered a gold standard. However, only 15% of published MS studies used any validation strategy and only 8% used independent validation (10).

In conclusion, covariate-adjusted models outperform correlations of any single MRI biomarker with clinical outcomes. These models are therefore likely more sensitive imaging outcomes. Additionally, by subtracting covariates, these models should more accurately reflect MS-specific effects on CNS tissue and thus might be more responsive to MS-targeted treatments.

This hypothesis should be tested in future clinical trials, especially when targeting older subjects with progressive MS who no longer form acute MS lesions. Current paper provides all equations for adjusting MRI volumes and list of variables in each model. The final models should be recreated from pre-treatment (baseline) data collected during clinical trial to compensate for the variance caused by differences in scanning protocols and volumetric analysis methods.

## Data availability statement

Raw data and R codes used for analyses are available in the Supplementary material.

## Ethics statement

The studies involving human participants were reviewed and approved by National Institutes of Health combined IRB. The patients/participants provided their written informed consent to participate in this study.

## Author contributions

YK and MV performed MRI scan processing, volumetric analyses, and maintained the cloud-based QMENTA platform to generate brain volumetric data. YK performed all analyses detailed in the paper, generated the figures, and contributed to writing the paper. PK exported all clinical scores used in correlation analyses and supervised in the development of GBM models. BB construed the project conceptually, guided and supervised all aspects of the study, and contributed to the writing of the paper and figure creations. All authors critically reviewed and edited the manuscript.

## Funding

The research was supported by the Intramural Research Program of the National Institute of Allergy and Infectious Diseases (NIAID), National Institutes of Health (NIH).

## Conflict of interest

The authors declare that the research was conducted in the absence of any commercial or financial relationships that could be construed as a potential conflict of interest.

## Publisher's note

All claims expressed in this article are solely those of the authors and do not necessarily represent those of their affiliated organizations, or those of the publisher, the editors and the reviewers. Any product that may be evaluated in this article, or claim that may be made by its manufacturer, is not guaranteed or endorsed by the publisher.
