# Supplementary material for: Confounder-adjusted MRI-based predictors of multiple sclerosis disability
Source: Front Radiol. 2022 Sep 13;2:971157. doi: 10.3389/fradi.2022.971157 (PMC10365278; doi:10.3389/fradi.2022.971157)
Supplement: Supplementary file 1 [file Data_Sheet_1.pdf]

SUPPLEMENTARY FIGURES

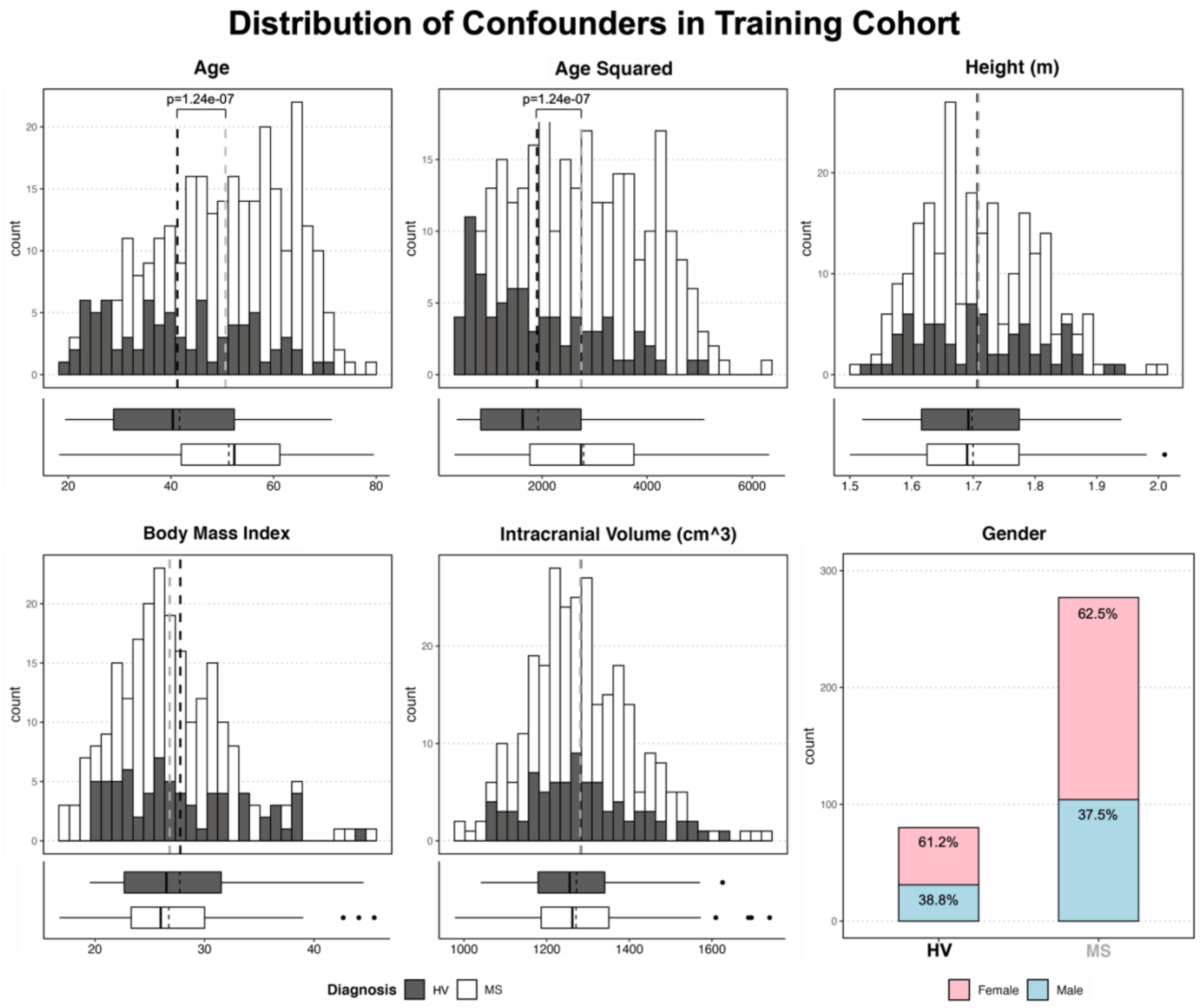

Figure S1. Differences in cohort characteristics between healthy volunteers (HV) and MS patients. Distribution of five continuous confounders is shown as histograms with HV cohort in black and MS cohort in while colors. The proportions and absolute numbers of males and females in HV and MS cohorts are displayed as a bar chart with females show in pink and males in blue. There are no significant differences between HV and MS cohort in all confounders except age (and age^2).

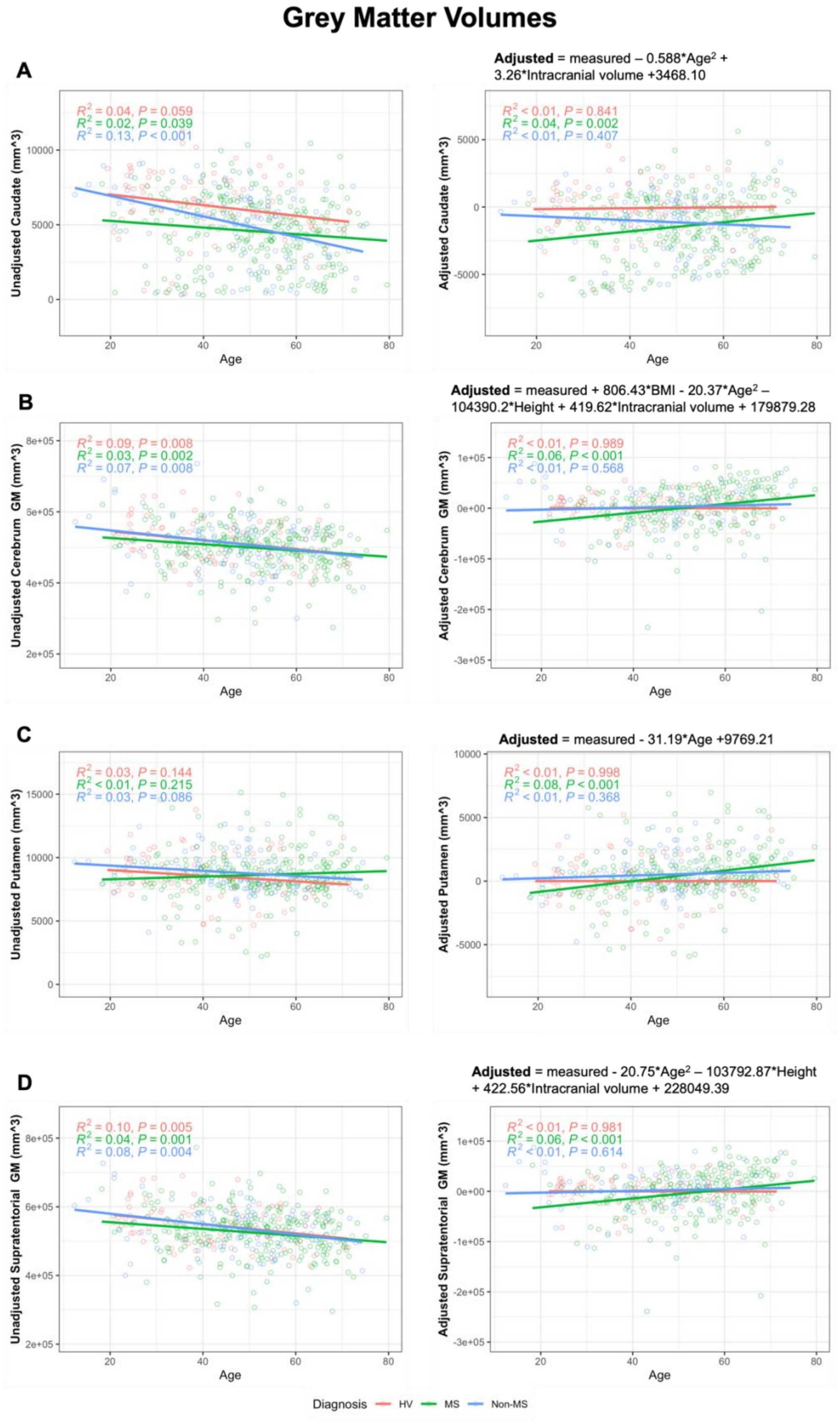
Grey Matter Volumes
A
Adjusted = measured – 0.588*Age2 + 3.26*Intracranial volume +3468.10
R2 = 0.04, P = 0.059
R2 = 0.02, P = 0.039
R2 = 0.13, P < 0.001
Unadjusted Caudate (mm^3)
R2 < 0.01, P = 0.841
R2 = 0.04, P = 0.002
R2 < 0.01, P = 0.407
Adjusted Caudate (mm^3)
Age
B
Adjusted = measured + 806.43*BMI - 20.37*Age2 – 104390.2*Height + 419.62*Intracranial volume + 179879.28
R2 = 0.09, P = 0.008
R2 = 0.03, P = 0.002
R2 = 0.07, P = 0.008
Unadjusted Cerebrum GM (mm^3)
R2 < 0.01, P = 0.989
R2 = 0.06, P < 0.001
R2 < 0.01, P = 0.568
Adjusted Cerebrum GM (mm^3)
Age
C
Adjusted = measured - 31.19*Age +9769.21
R2 = 0.03, P = 0.144
R2 < 0.01, P = 0.215
R2 = 0.03, P = 0.086
Unadjusted Putamen (mm^3)
R2 < 0.01, P = 0.998
R2 = 0.08, P < 0.001
R2 < 0.01, P = 0.368
Adjusted Putamen (mm^3)
Age
D
Adjusted = measured - 20.75*Age2 – 103792.87*Height + 422.56*Intracranial volume + 228049.39
R2 = 0.10, P = 0.005
R2 = 0.04, P = 0.001
R2 = 0.08, P = 0.004
Unadjusted Supratentorial GM (mm^3)
R2 < 0.01, P = 0.981
R2 = 0.06, P < 0.001
R2 < 0.01, P = 0.614
Adjusted Supratentorial GM (mm^3)
Age
Diagnosis HV MS Non-MS

Figure S2. Grey matter volumes with residual variance with age within training cohort. Left panels all show unadjusted grey matter volumes while right panels show confounder-adjusted grey matter volumes. Red line and points indicate HV cohort while blue line and points indicate MS cohort. Adjustment equations respective to MRI feature are shown on top of the plot showing adjusted MRI volumes.

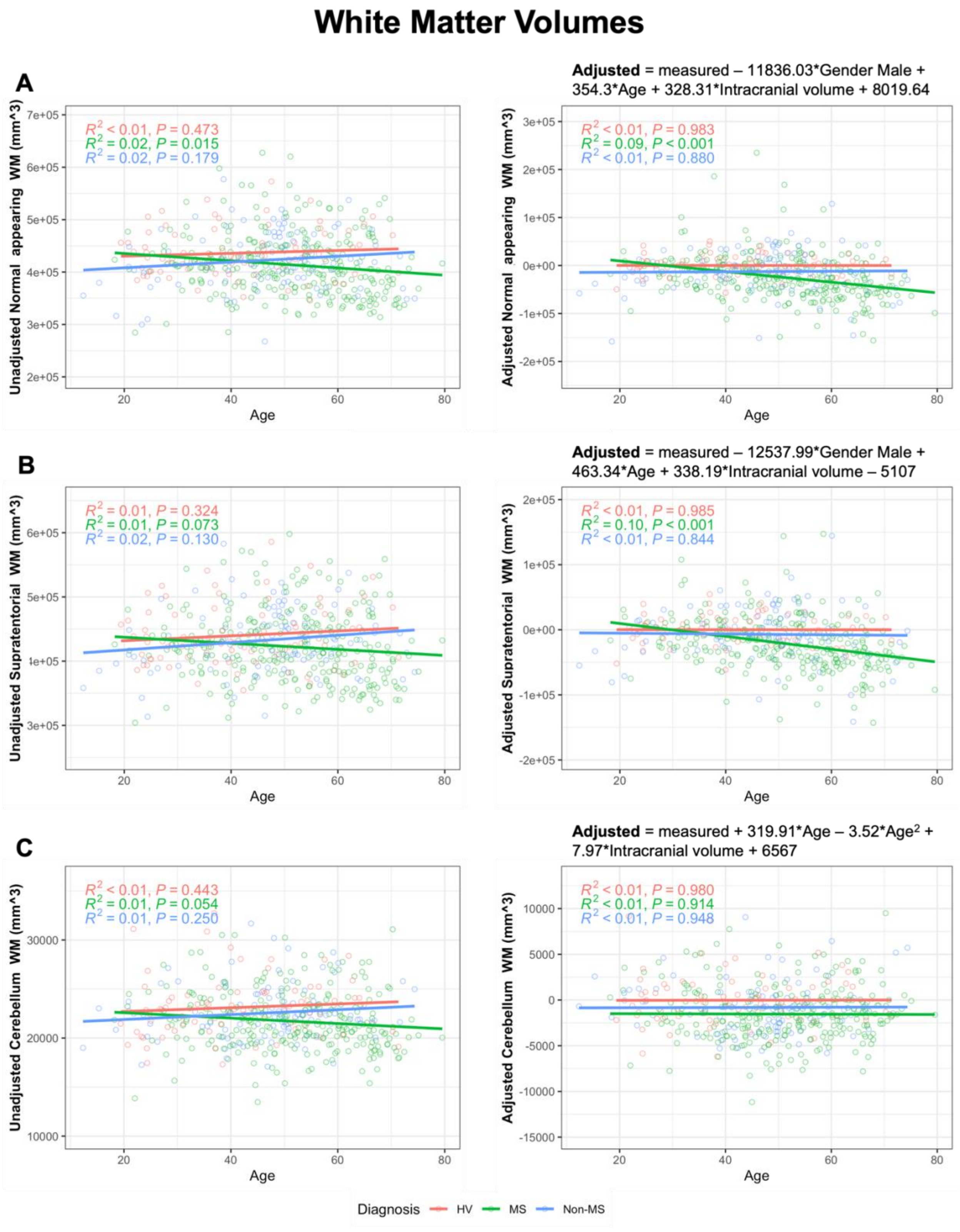
White Matter Volumes
A
Adjusted = measured – 11836.03*Gender Male + 354.3*Age + 328.31*Intracranial volume + 8019.64
R^2 < 0.01, P = 0.473
R^2 = 0.02, P = 0.015
R^2 = 0.02, P = 0.179
Unadjusted Normal appearing WM (mm^3)
R^2 < 0.01, P = 0.983
R^2 = 0.09, P < 0.001
R^2 < 0.01, P = 0.880
Adjusted Normal appearing WM (mm^3)
Age
B
Adjusted = measured – 12537.99*Gender Male + 463.34*Age + 338.19*Intracranial volume – 5107
R^2 = 0.01, P = 0.324
R^2 = 0.01, P = 0.073
R^2 = 0.02, P = 0.130
Unadjusted Supratentorial WM (mm^3)
R^2 < 0.01, P = 0.985
R^2 = 0.10, P < 0.001
R^2 < 0.01, P = 0.844
Adjusted Supratentorial WM (mm^3)
Age
C
Adjusted = measured + 319.91*Age – 3.52*Age^2 + 7.97*Intracranial volume + 6567
R^2 < 0.01, P = 0.443
R^2 = 0.01, P = 0.054
R^2 = 0.01, P = 0.250
Unadjusted Cerebellum WM (mm^3)
R^2 < 0.01, P = 0.980
R^2 < 0.01, P = 0.914
R^2 < 0.01, P = 0.948
Adjusted Cerebellum WM (mm^3)
Age
Diagnosis HV MS Non-MS

Figure S3. Left panels all show unadjusted white matter volumes while right panels show confounder-adjusted white matter volumes. Red line and points indicate HV cohort while blue line and points indicate MS cohort. Adjustment equations respective to MRI feature are shown on top of the plot showing adjusted MRI volumes.

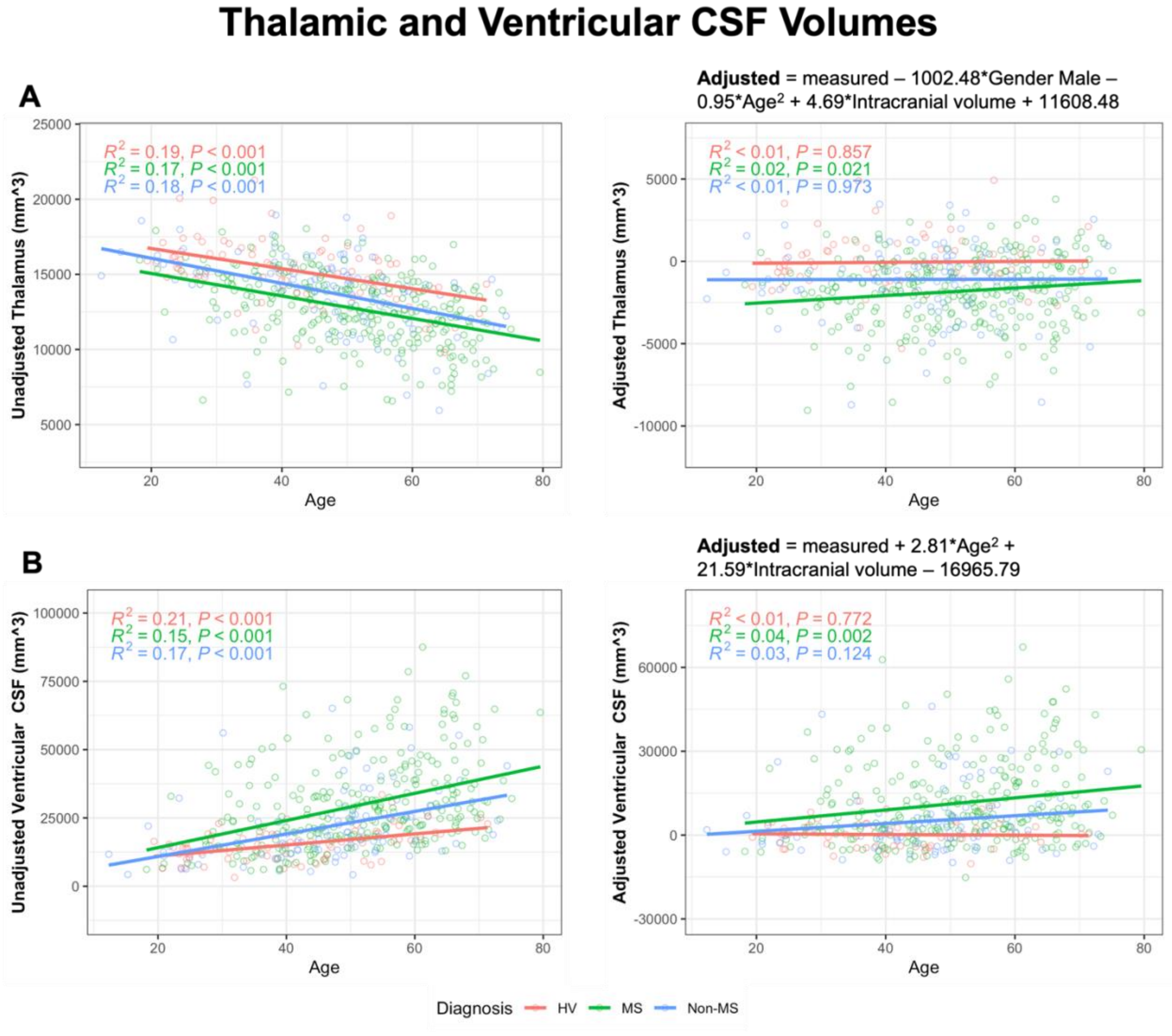

Figure S4. Left panels all show unadjusted thalamic and ventricular CSF volumes while right panels show confounder-adjusted volumes. Red line and points indicate HV cohort while blue line and points indicate MS cohort. Adjustment equations respective to MRI feature are shown on top of the plot showing adjusted MRI volumes.

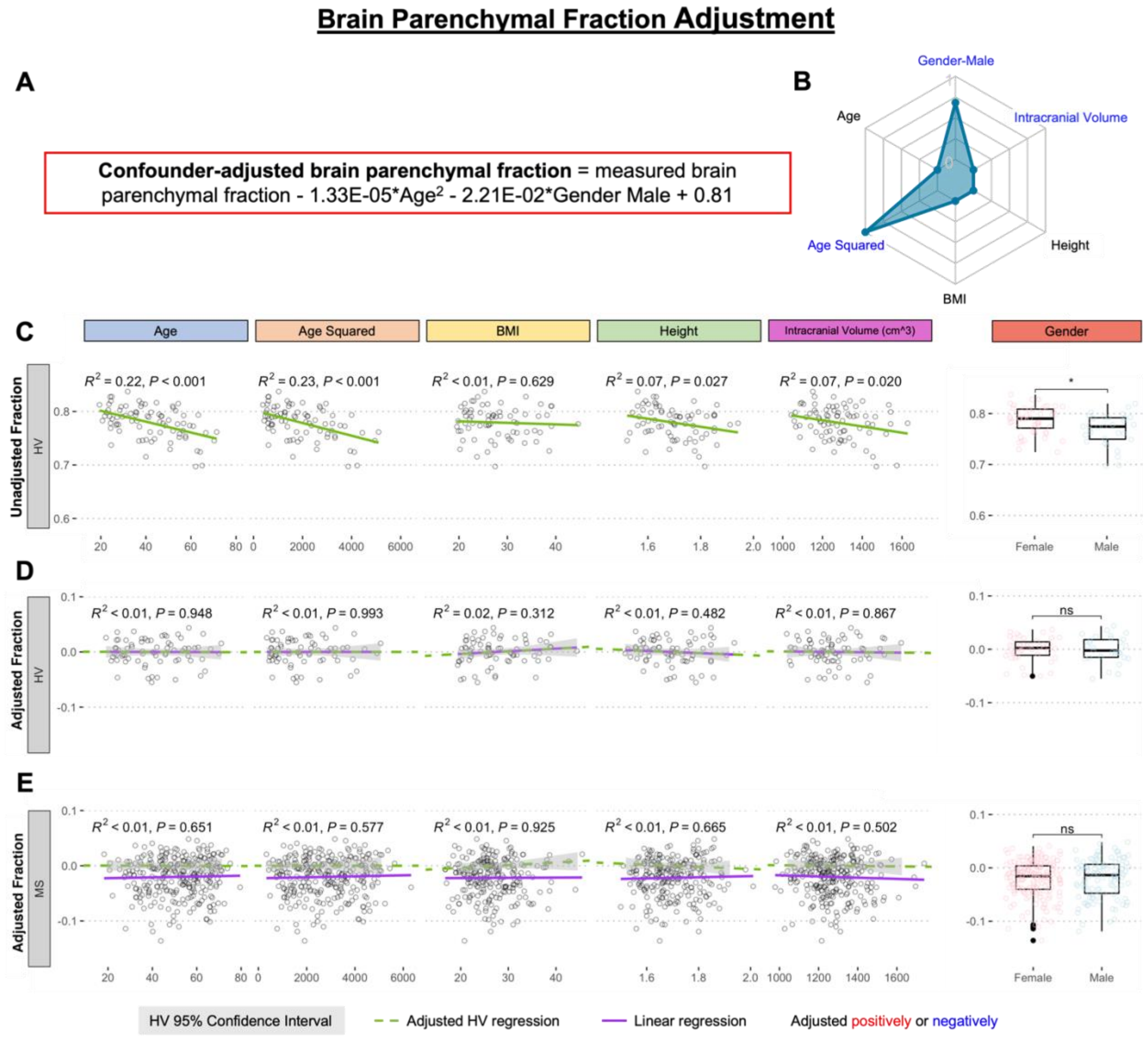

Figure S5. Adjustment of single MRI biomarker (i.e., brain parenchymal fraction) for six physiological confounders. A. The final equation for the confounder-adjusted brain parenchymal fraction is shown at the top in red outline. This equation was derived from multiple linear regression models as described in the Method section. **B.** In the top right corner is resulting radar chart that show proportional weights of the applied confounder adjustment, with confounders with lowest weights (i.e., the innermost circle) representing zeros. **C.** Univariate linear regression models between each tested confounder on x-axis (first 5 graphs) or gender

(sixth graph) and measured brain parenchymal fraction on the y-axis in 80 healthy volunteers (HV). **D.** Same univariate regressions in the HV cohort after applying adjustment formula show no remaining effect of confounders. **E.** Applying HV-derived adjustment formula to MS cohort shows no residual effect.

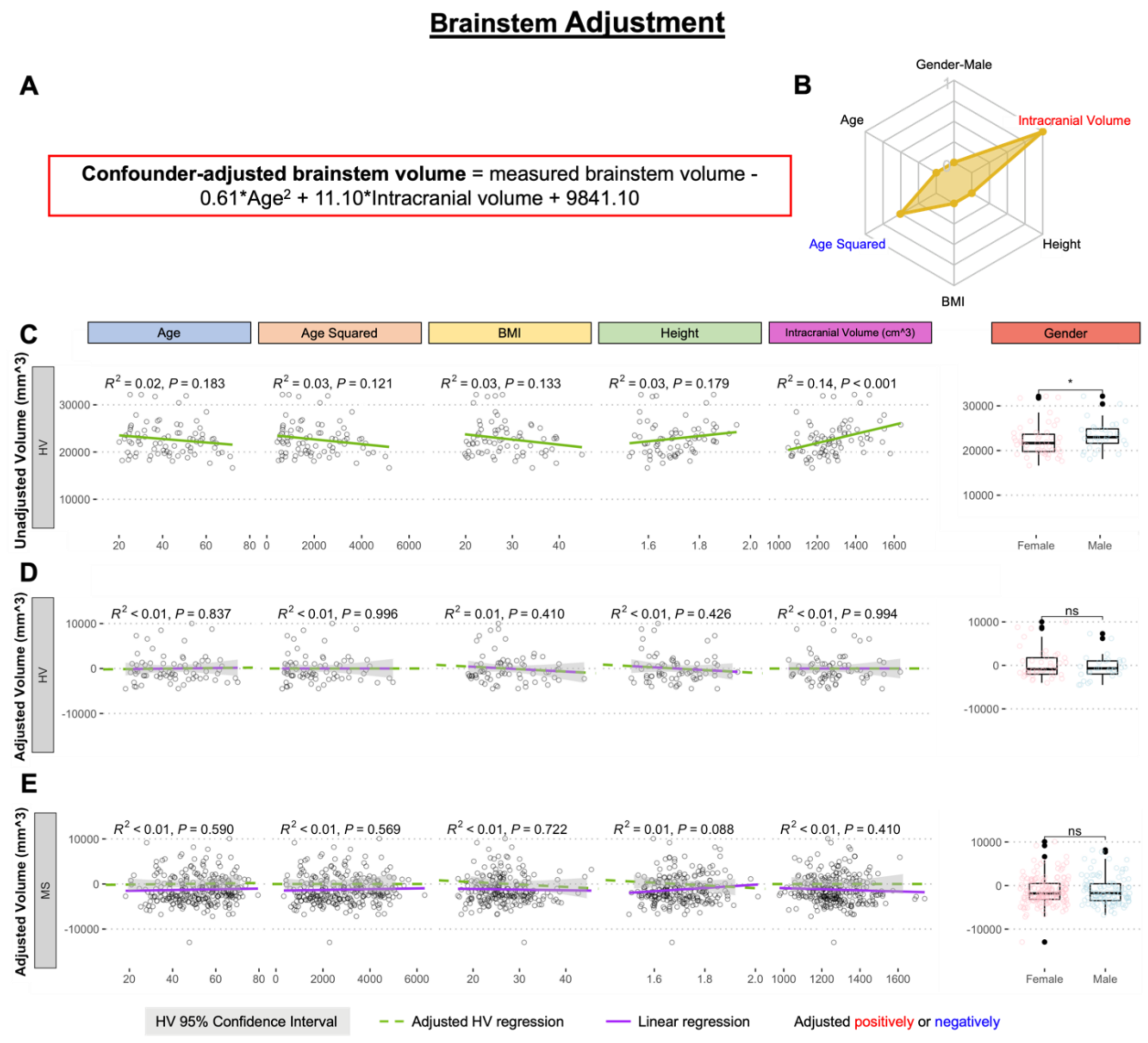

Figure S6. Adjustment of single MRI biomarker (i.e., brainstem volume) for six physiological confounders. A. The final equation for the confounder-adjusted brainstem volume is shown at the top in red outline. This equation was derived from multiple linear regression models as described in the Method section. **B.** In the top right corner is resulting radar chart that show proportional weights of the applied confounder adjustment, with confounders with lowest weights (i.e., the innermost circle) representing zeros. **C.** Univariate linear regression models between each tested confounder on x-axis (first 5 graphs) or gender (sixth graph) and measured

brainstem volume on the y-axis in 80 healthy volunteers (HV). **D.** Same univariate regressions in the HV cohort after applying adjustment formula show no remaining effect of confounders. **E.** Applying HV-derived adjustment formula to MS cohort resulted in no residual effect.

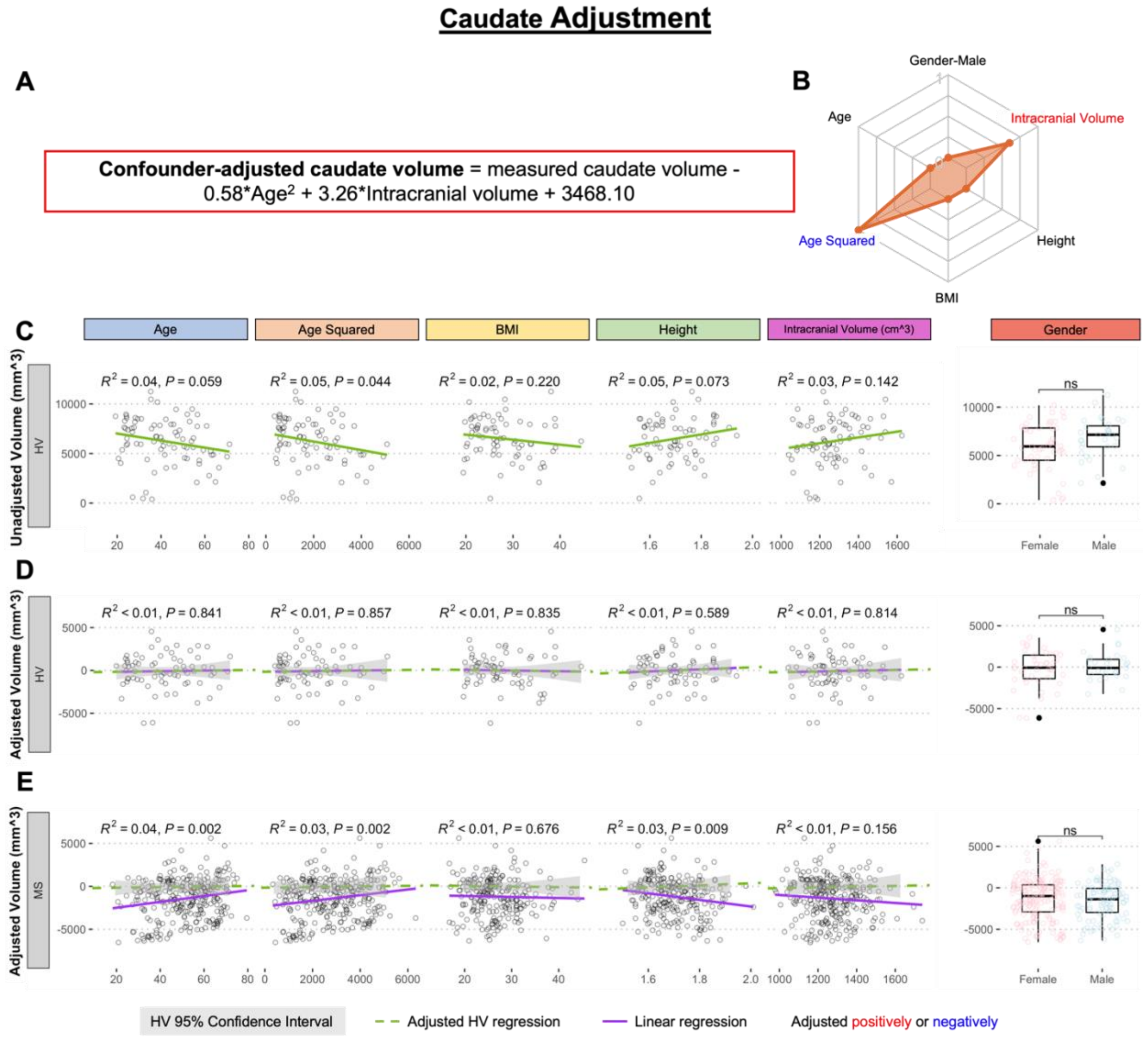

Figure S7. Adjustment of single MRI biomarker (i.e., caudate volume) for six physiological confounders. A. The final equation for the confounder-adjusted caudate volume is shown at the top in red outline. This equation was derived from multiple linear regression models as described in the Method section. **B.** In the top right corner is resulting radar chart that show proportional weights of the applied confounder adjustment, with confounders with lowest weights (i.e., the innermost circle) representing zeros. **C.** Univariate linear regression models between each tested confounder on x-axis (first 5 graphs) or gender (sixth graph) and measured caudate volume on

the y-axis in 80 healthy volunteers (HV). **D.** Same univariate regressions in the HV cohort after applying adjustment formula show no remaining effect of confounders. **E.** Applying HV-derived adjustment formula to MS cohort shows residual effect of age and age^2, when considering Bonferroni adjustment for multiple comparisons (i.e., p<0.05/12 = p<0.004).

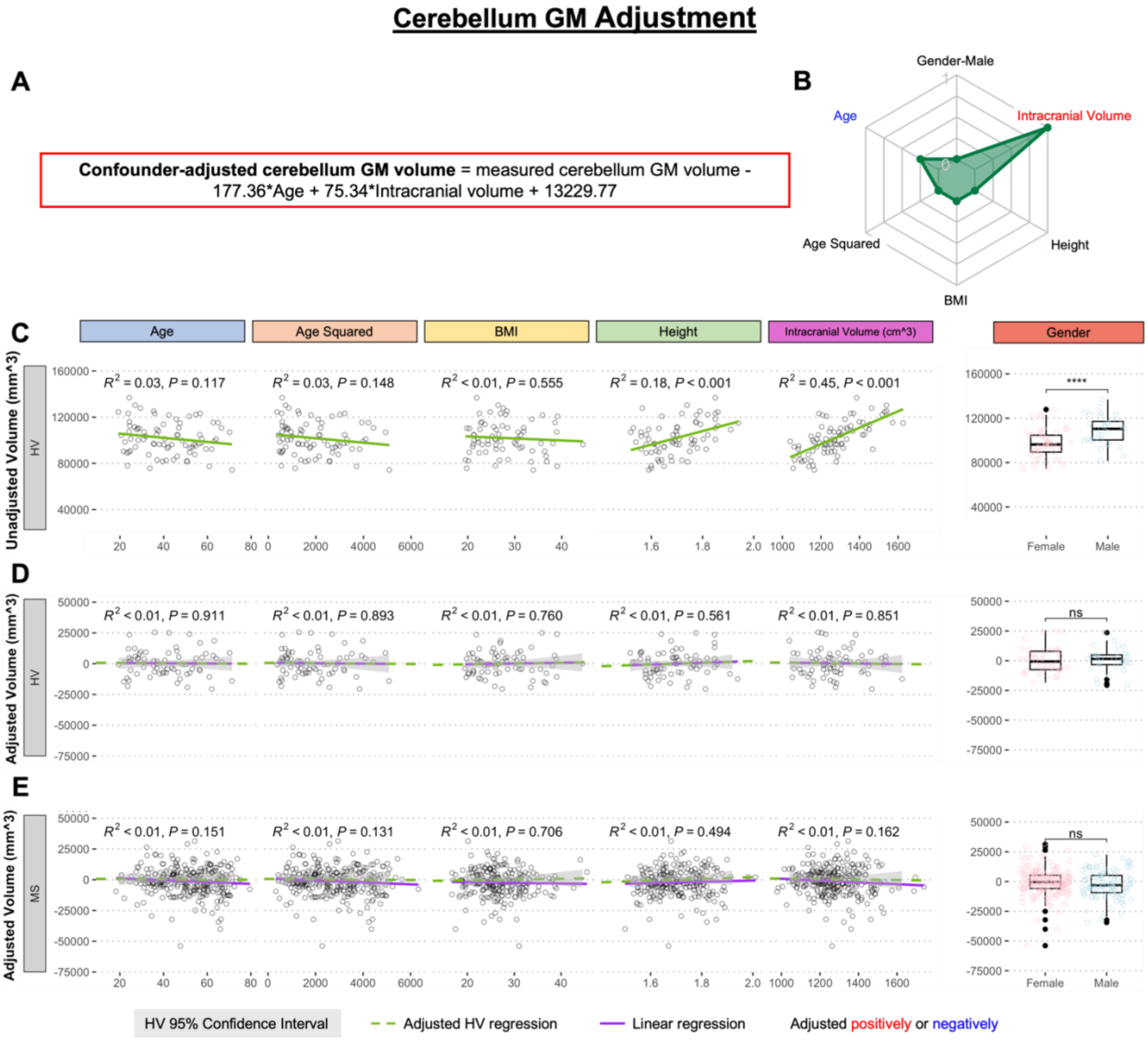

Figure S8. Adjustment of single MRI biomarker (i.e., cerebellum GM volume) for six physiological confounders. A. The final equation for the confounder-adjusted cerebellum GM volume is shown at the top in red outline. This equation was derived from multiple linear

regression models as described in the Method section. **B.** In the top right corner is resulting radar chart that show proportional weights of the applied confounder adjustment, with confounders with lowest weights (i.e., the innermost circle) representing zeros. **C.** Univariate linear regression models between each tested confounder on x-axis (first 5 graphs) or gender (sixth graph) and measured cerebellum GM volume on the y-axis in 80 healthy volunteers (HV). **D.** Same univariate regressions in the HV cohort after applying adjustment formula show no remaining effect of confounders. **E.** Applying HV-derived adjustment formula to MS cohort resulted in no residual effect.

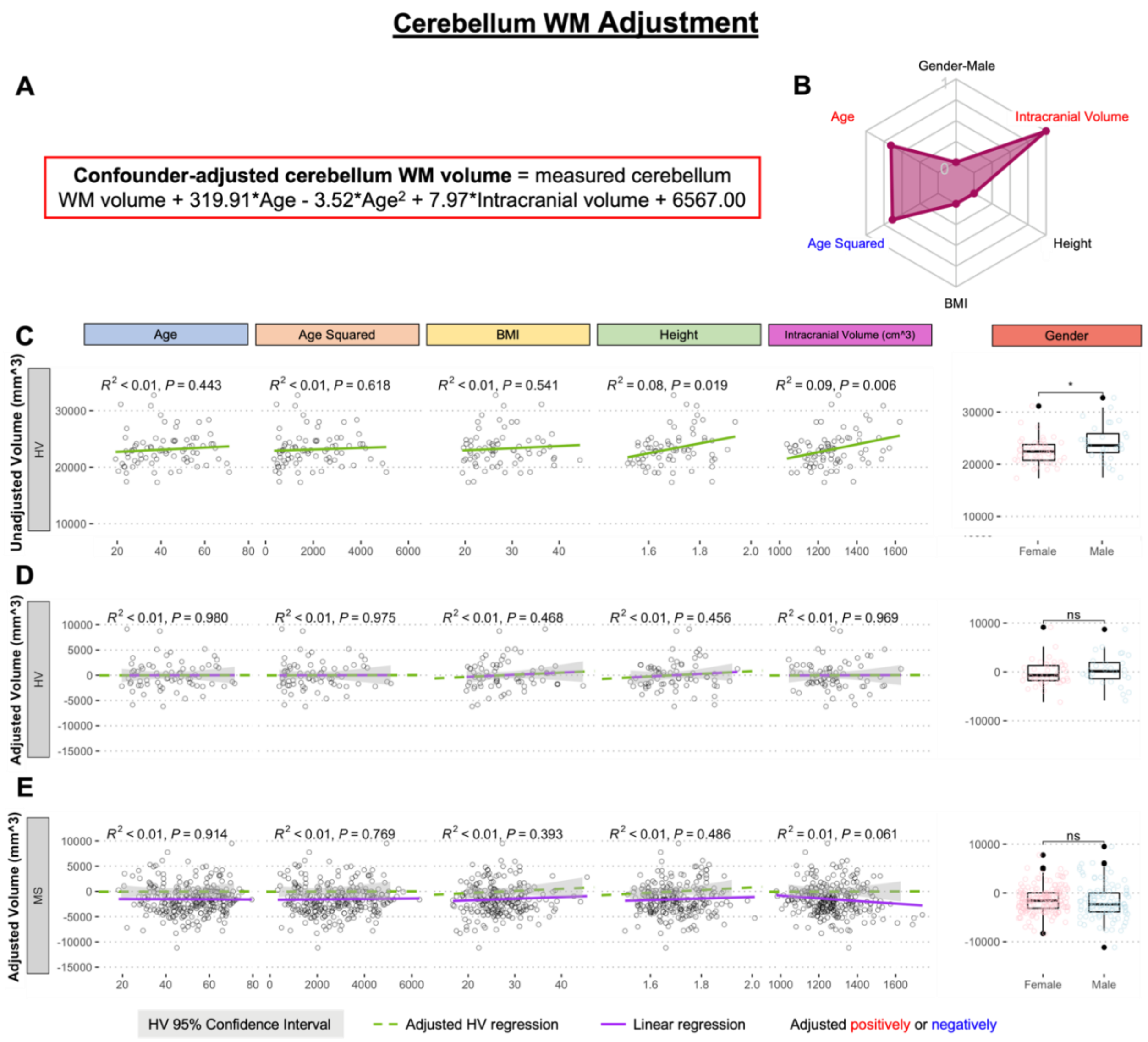

Figure S9. Adjustment of single MRI biomarker (i.e., cerebellum WM volume) for six physiological confounders. A. The final equation for the confounder-adjusted cerebellum WM volume is shown at the top in red outline. This equation was derived from multiple linear regression models as described in the Method section. **B.** In the top right corner is resulting radar chart that show proportional weights of the applied confounder adjustment, with confounders with lowest weights (i.e., the innermost circle) representing zeros. **C.** Univariate linear regression models between each tested confounder on x-axis (first 5 graphs) or gender (sixth graph) and

measured cerebellum WM volume on the y-axis in 80 healthy volunteers (HV). **D.** Same univariate regressions in the HV cohort after applying adjustment formula show no remaining effect of confounders. **E.** Applying HV-derived adjustment formula to MS cohort resulted in no residual effect.

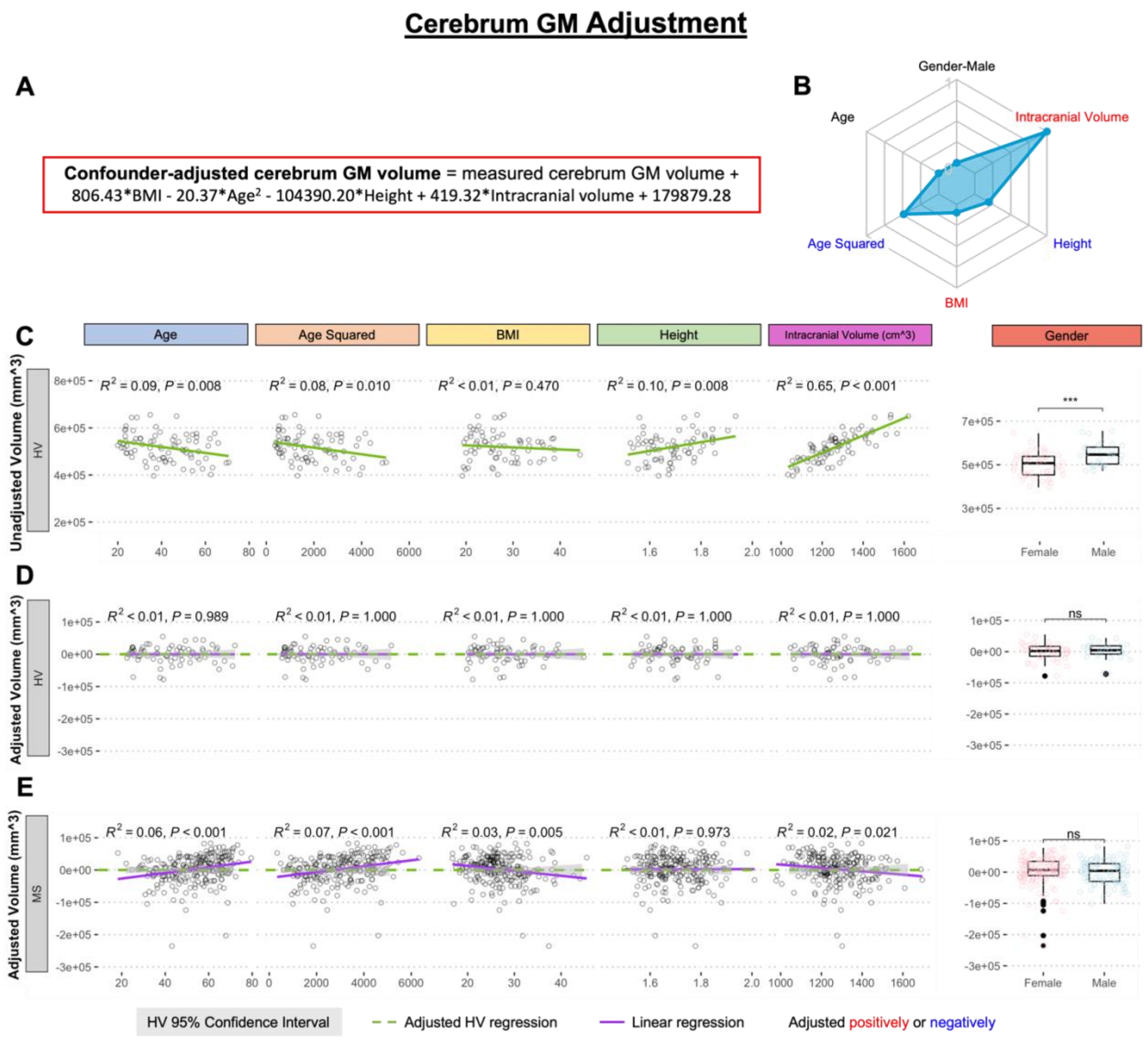

Figure S10. Adjustment of single MRI biomarker (i.e., cerebrum GM volume) for six physiological confounders. A. The final equation for the confounder-adjusted cerebrum GM volume is shown at the top in red outline. This equation was derived from multiple linear regression models as described in the Method section. **B.** In the top right corner is resulting radar chart that show proportional weights of the applied confounder adjustment, with confounders with lowest weights (i.e., the innermost circle) representing zeros. **C.** Univariate linear regression models between each tested confounder on x-axis (first 5 graphs) or gender (sixth graph) and

measured cerebrum GM volume on the y-axis in 80 healthy volunteers (HV). **D.** Same univariate regressions in the HV cohort after applying adjustment formula show no remaining effect of confounders. **E.** Applying HV-derived adjustment formula to MS cohort shows residual effect of age and age^2 when considering Bonferroni adjustment for multiple comparisons (i.e., $p<0.05/12 = p<0.004$).

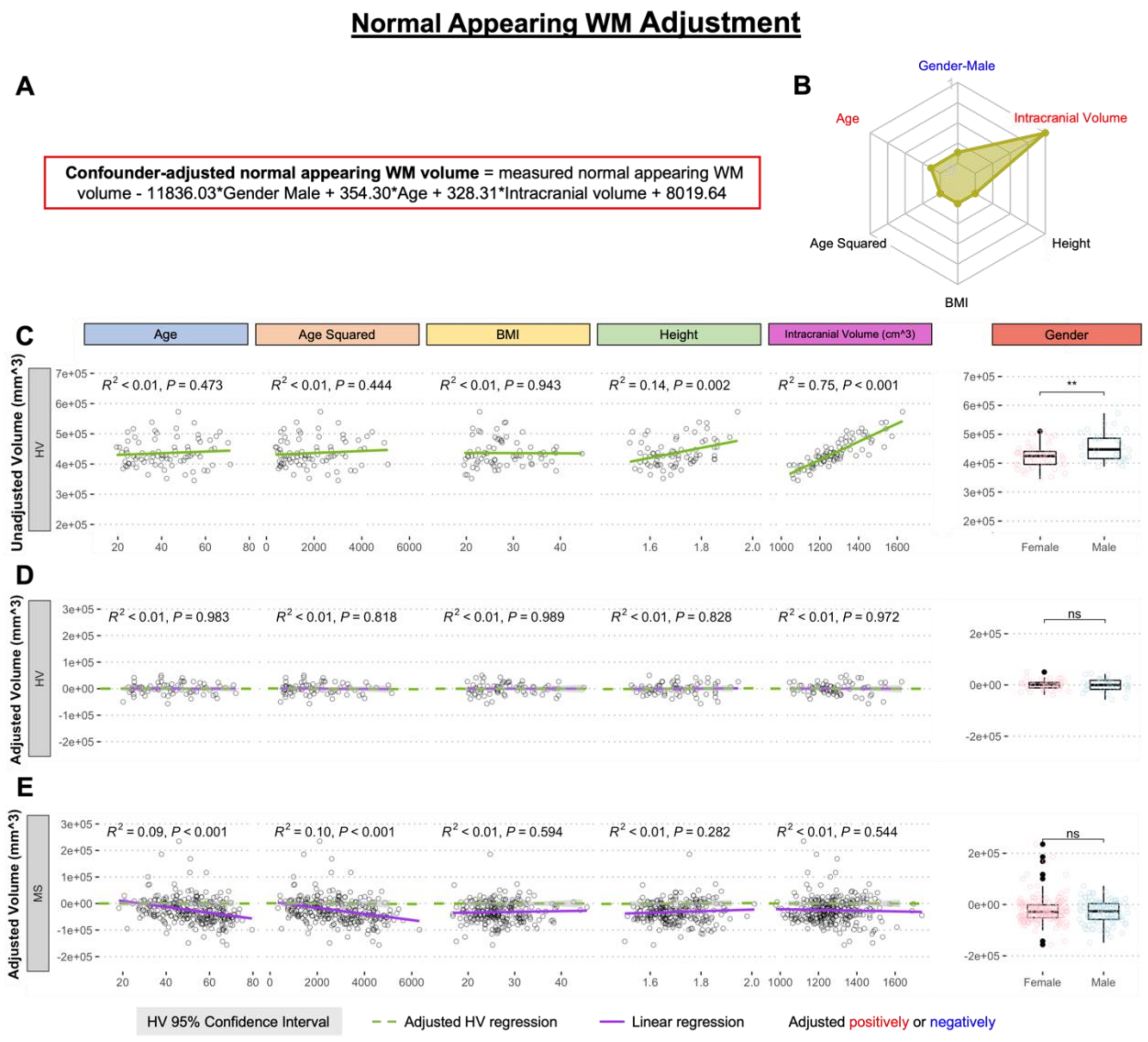

Figure S11. Adjustment of single MRI biomarker (i.e., normal-appearing WM volume) for six physiological confounders. A. The final equation for the confounder-adjusted normal-appearing WM volume is shown at the top in red outline. This equation was derived from multiple linear regression models as described in the Method section. **B.** In the top right corner is resulting radar chart that show proportional weights of the applied confounder adjustment, with confounders with lowest weights (i.e., the innermost circle) representing zeros. **C.** Univariate linear regression models between each tested confounder on x-axis (first 5 graphs) or gender

(sixth graph) and measured normal-appearing WM volume on the y-axis in 80 healthy volunteers (HV). **D.** Same univariate regressions in the HV cohort after applying adjustment formula show no remaining effect of confounders. **E.** Applying HV-derived adjustment formula to MS cohort shows residual effect of age and age^2.

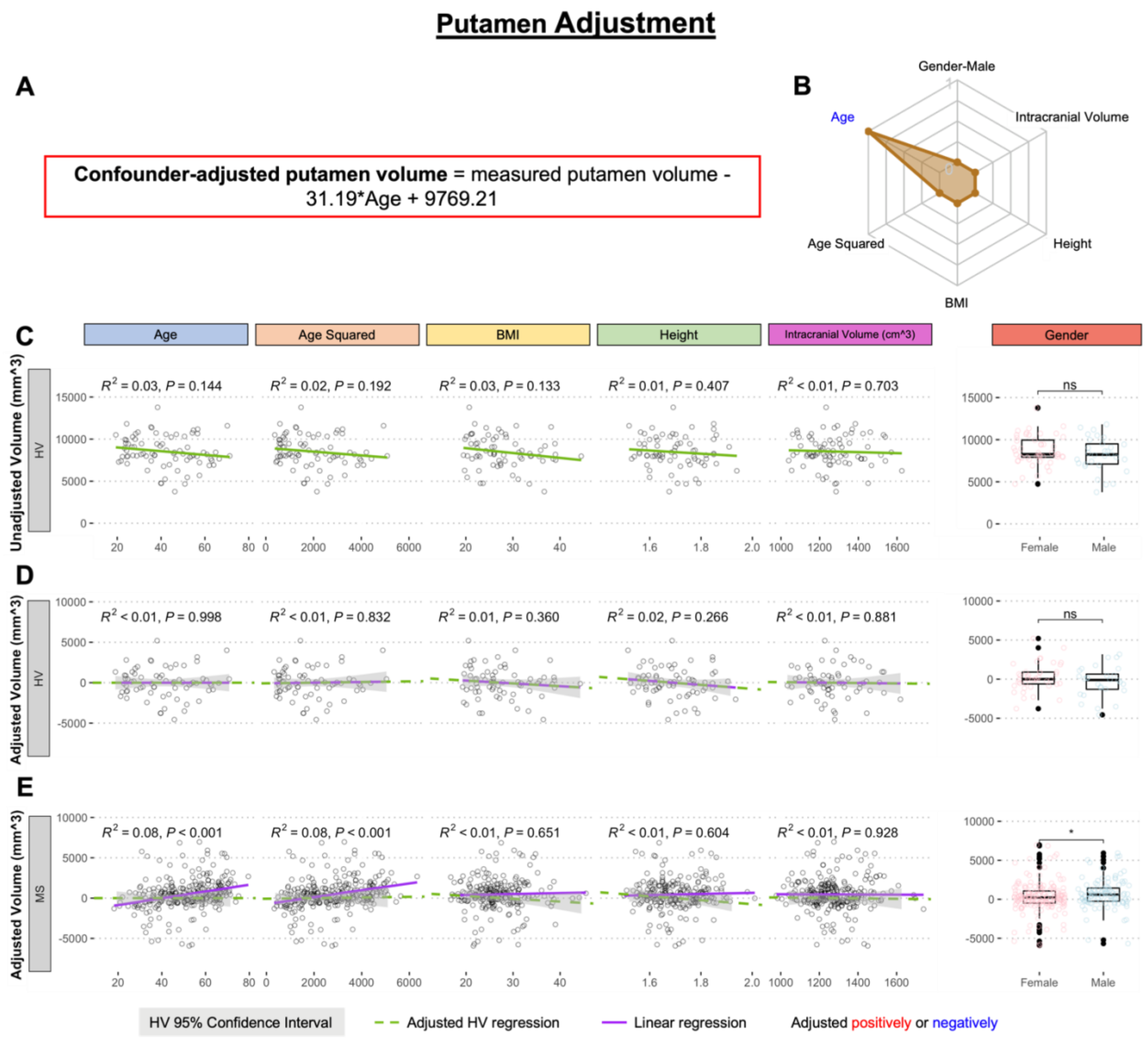

Figure S12. Adjustment of single MRI biomarker (i.e., putamen volume) for six physiological confounders. A. The final equation for the confounder-adjusted putamen volume is shown at the top in red outline. This equation was derived from multiple linear regression models as described in the Method section. **B.** In the top right corner is resulting radar chart that show proportional weights of the applied confounder adjustment, with confounders with lowest weights (i.e., the innermost circle) representing zeros. **C.** Univariate linear regression models between each tested confounder on x-axis (first 5 graphs) or gender (sixth graph) and measured

putamen volume on the y-axis in 80 healthy volunteers (HV). **D.** Same univariate regressions in the HV cohort after applying adjustment formula show no remaining effect of confounders. **E.** Applying HV-derived adjustment formula to MS cohort shows residual effect of age and age^2, when considering Bonferroni adjustment for multiple comparisons (i.e., $p<0.05/12 = p<0.004$).

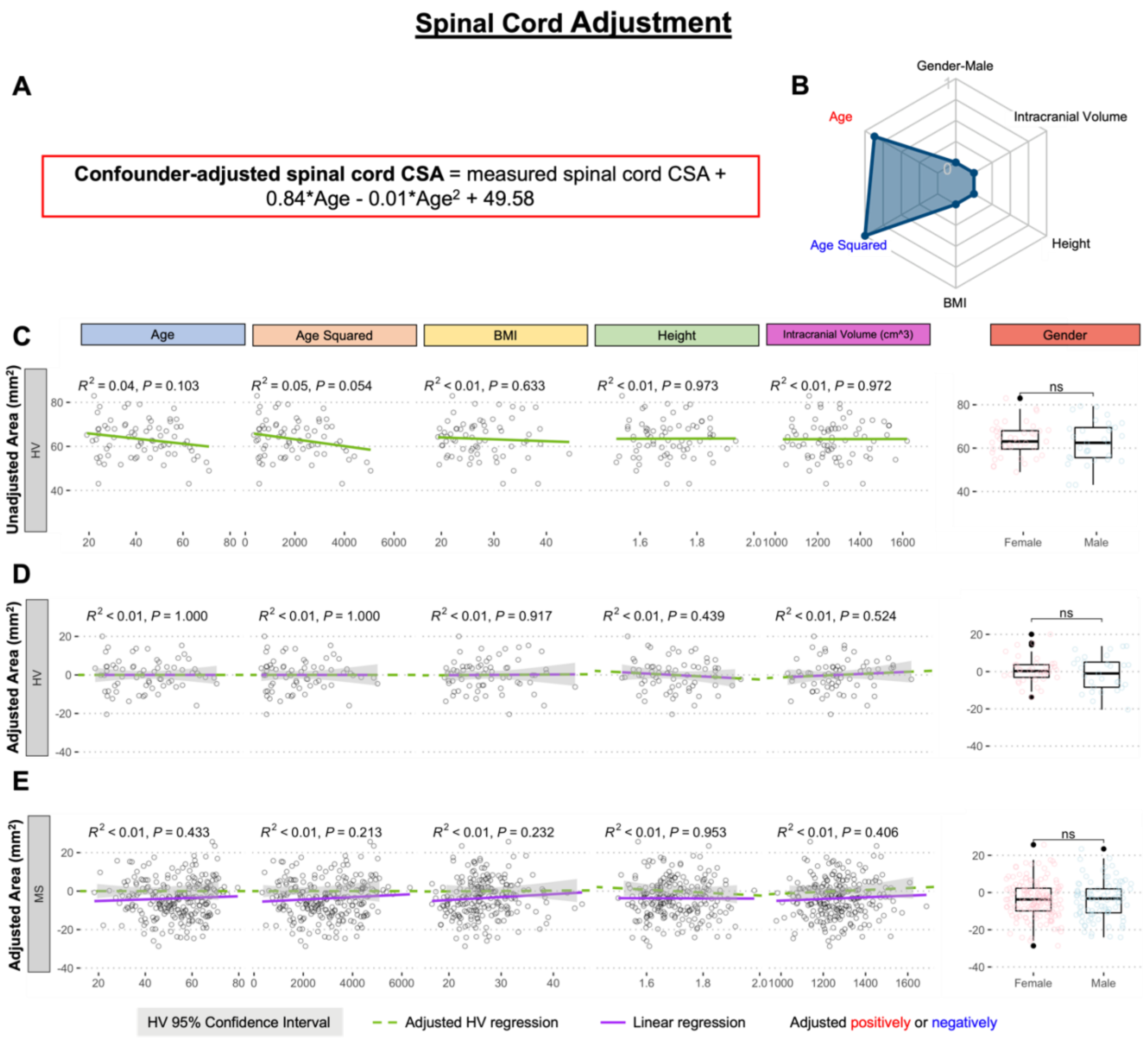

Figure S13. Adjustment of single MRI biomarker (i.e., spinal cord average cross section area) for six physiological confounders. A. The final equation for the confounder-adjusted spinal cord cross section area (CSA) is shown at the top in red outline. This equation was derived from multiple linear regression models as described in the Method section. **B.** In the top right corner is resulting radar chart that show proportional weights of the applied confounder adjustment, with confounders with lowest weights (i.e., the innermost circle) representing zeros. **C.** Univariate linear regression models between each tested confounder on x-axis (first 5 graphs) or gender (sixth graph) and measured spinal cord CSA on the y-axis in 80 healthy volunteers

(HV). **D.** Same univariate regressions in the HV cohort after applying adjustment formula show no remaining effect of confounders. **E.** Applying HV-derived adjustment formula to MS cohort resulted in no residual effect.

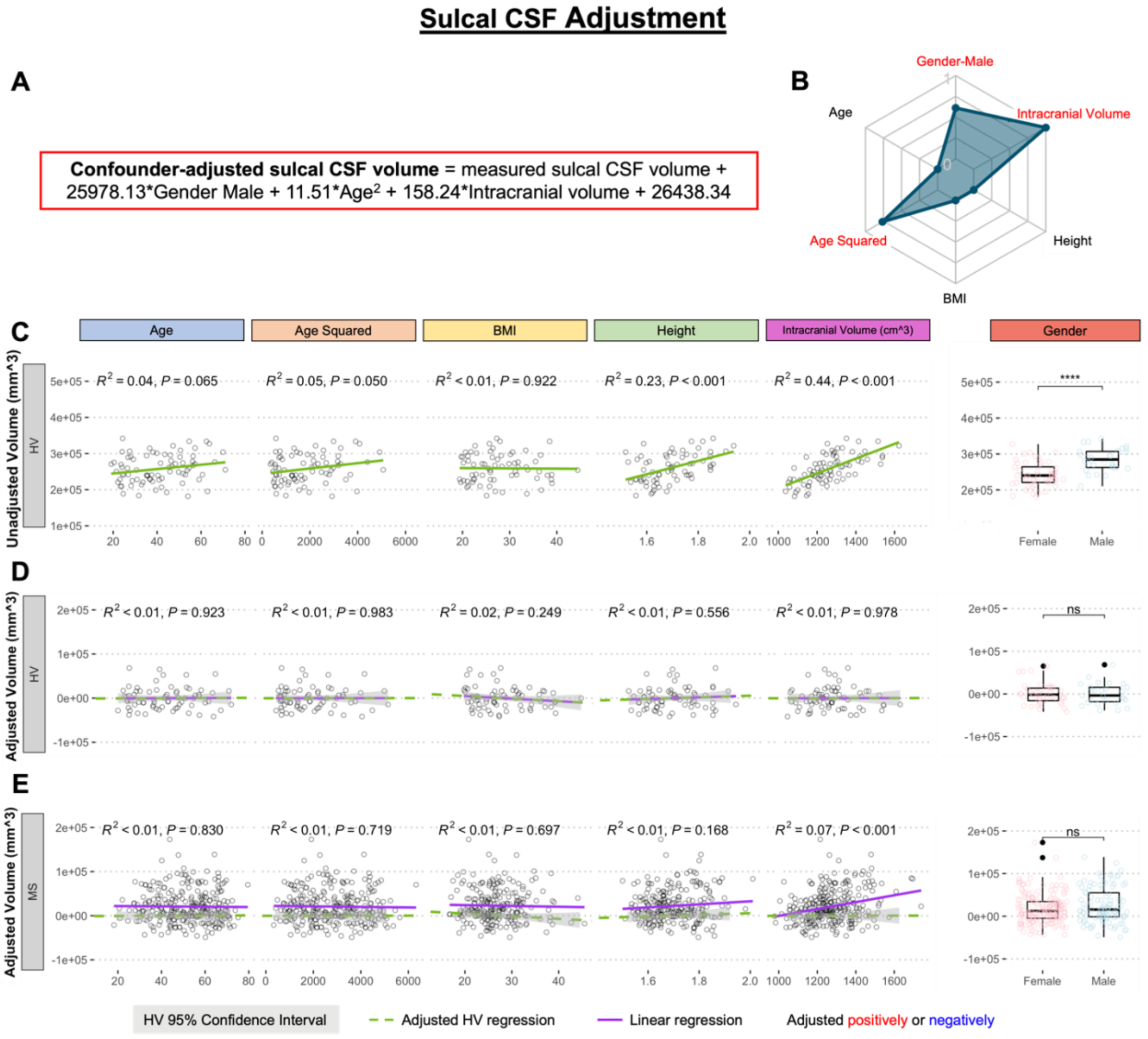

Figure S14. Adjustment of single MRI biomarker (i.e., sulcal CSF volume) for six physiological confounders. A. The final equation for the confounder-adjusted sulcal CSF volume is shown at the top in red outline. This equation was derived from multiple linear regression models as described in the Method section. **B.** In the top right corner is resulting radar chart that show proportional weights of the applied confounder adjustment, with confounders with lowest weights (i.e., the innermost circle) representing zeros. **C.** Univariate linear regression models between each tested confounder on x-axis (first 5 graphs) or gender (sixth graph) and

measured sulcal CSF volume on the y-axis in 80 healthy volunteers (HV). **D.** Same univariate regressions in the HV cohort after applying adjustment formula show no remaining effect of confounders. **E.** Applying HV-derived adjustment formula to MS cohort shows residual effect of intracranial volume.

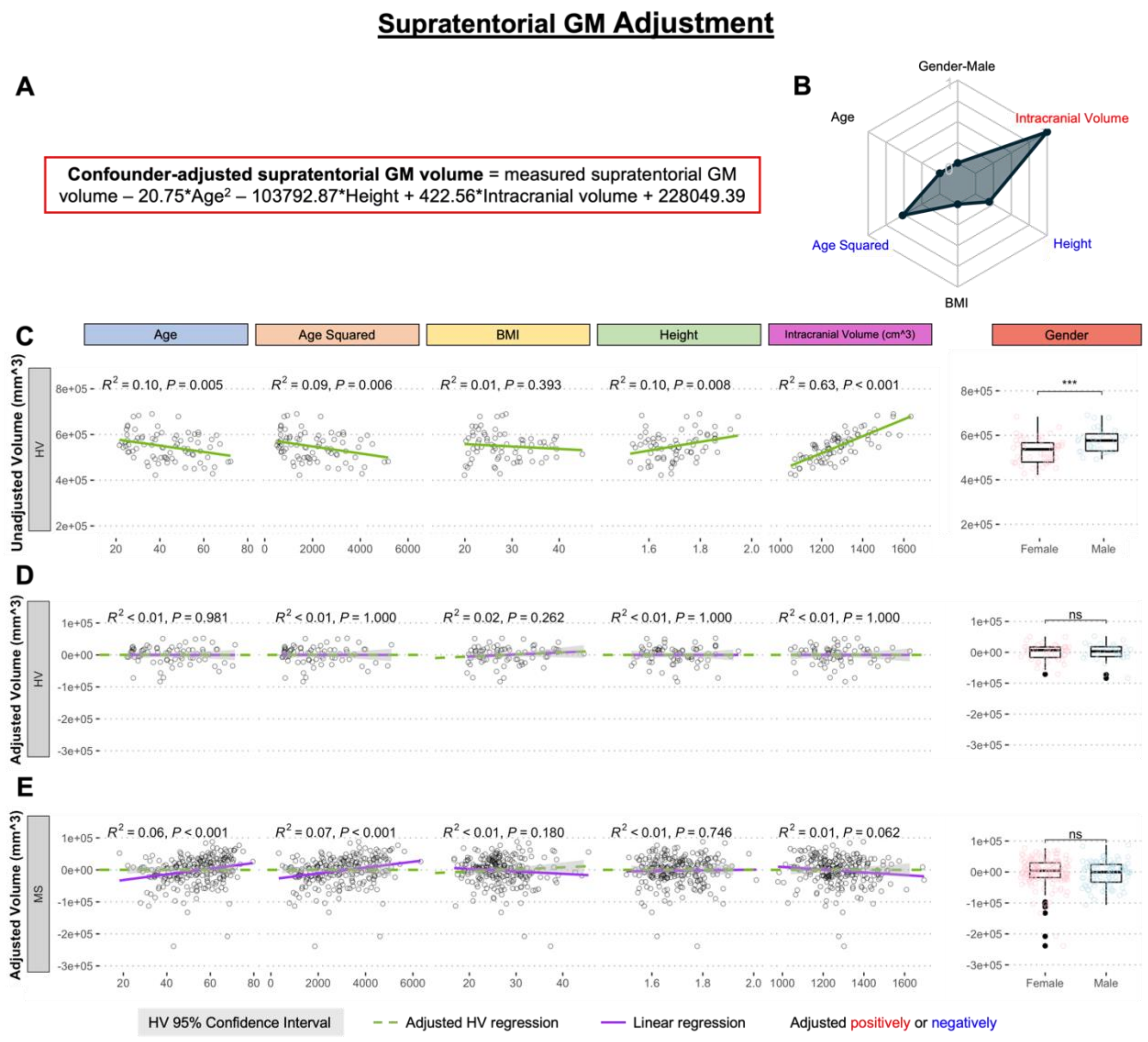

Figure S15. Adjustment of single MRI biomarker (i.e., supratentorial GM volume) for six physiological confounders. A. The final equation for the confounder-adjusted supratentorial GM volume is shown at the top in red outline. This equation was derived from multiple linear regression models as described in the Method section. **B.** In the top right corner is resulting radar chart that show proportional weights of the applied confounder adjustment, with confounders with lowest weights (i.e., the innermost circle) representing zeros. **C.** Univariate linear regression models between each tested confounder on x-axis (first 5 graphs) or gender (sixth graph) and

measured supratentorial GM volume on the y-axis in 80 healthy volunteers (HV). **D.** Same univariate regressions in the HV cohort after applying adjustment formula show no remaining effect of confounders. **E.** Applying HV-derived adjustment formula to MS cohort shows residual effect of age and age^2.

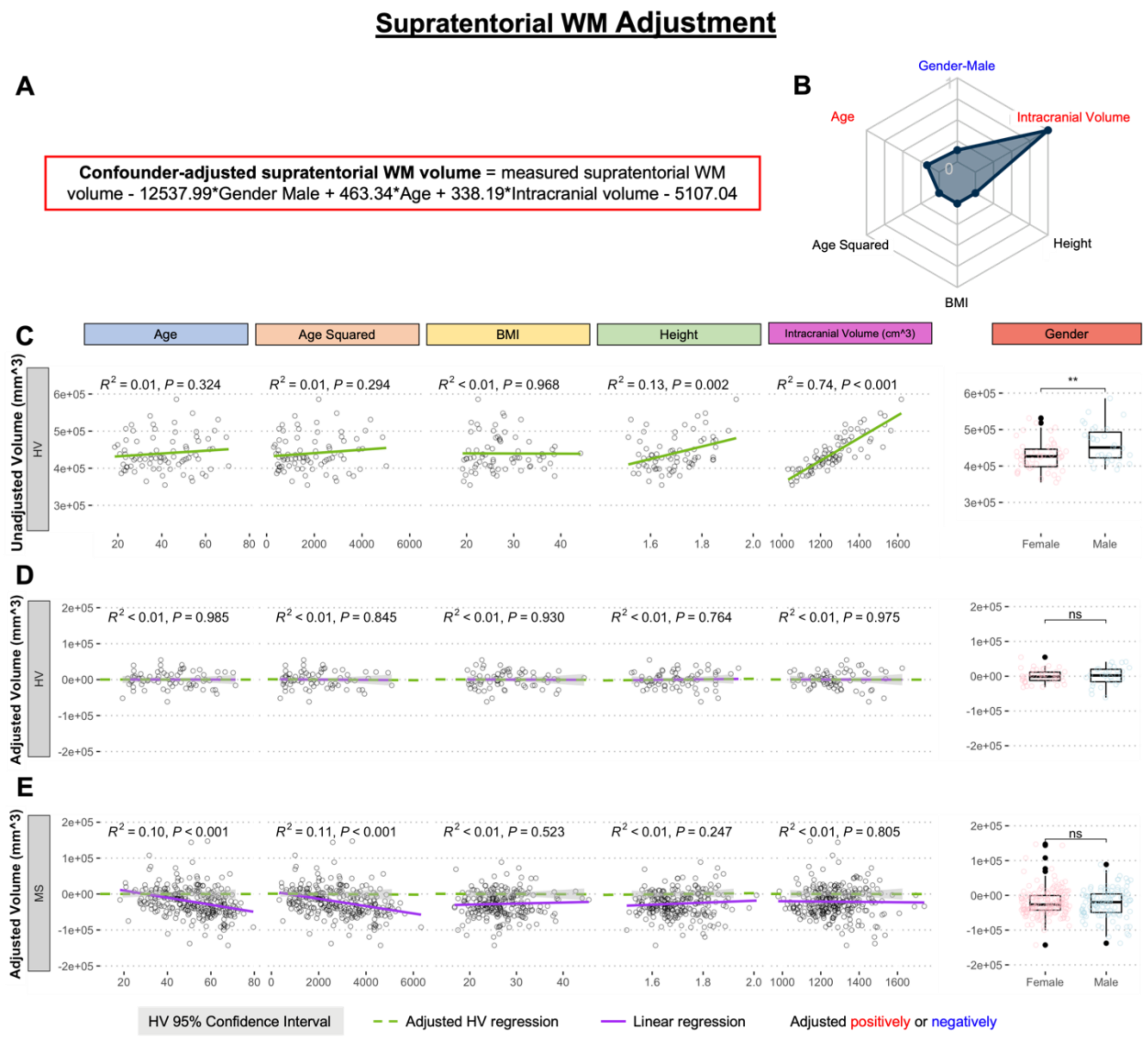

Figure S16. Adjustment of single MRI biomarker (i.e., supratentorial WM volume) for six physiological confounders. A. The final equation for the confounder-adjusted supratentorial WM volume is shown at the top in red outline. This equation was derived from multiple linear regression models as described in the Method section. **B.** In the top right corner is resulting radar chart that show proportional weights of the applied confounder adjustment, with confounders with lowest weights (i.e., the innermost circle) representing zeros. **C.** Univariate linear regression models between each tested confounder on x-axis (first 5 graphs) or gender (sixth graph) and

measured supratentorial WM volume on the y-axis in 80 healthy volunteers (HV). **D.** Same univariate regressions in the HV cohort after applying adjustment formula show no remaining residual effect of confounders. **E.** Applying HV-derived adjustment formula to MS cohort shows residual effect of age and age^2.

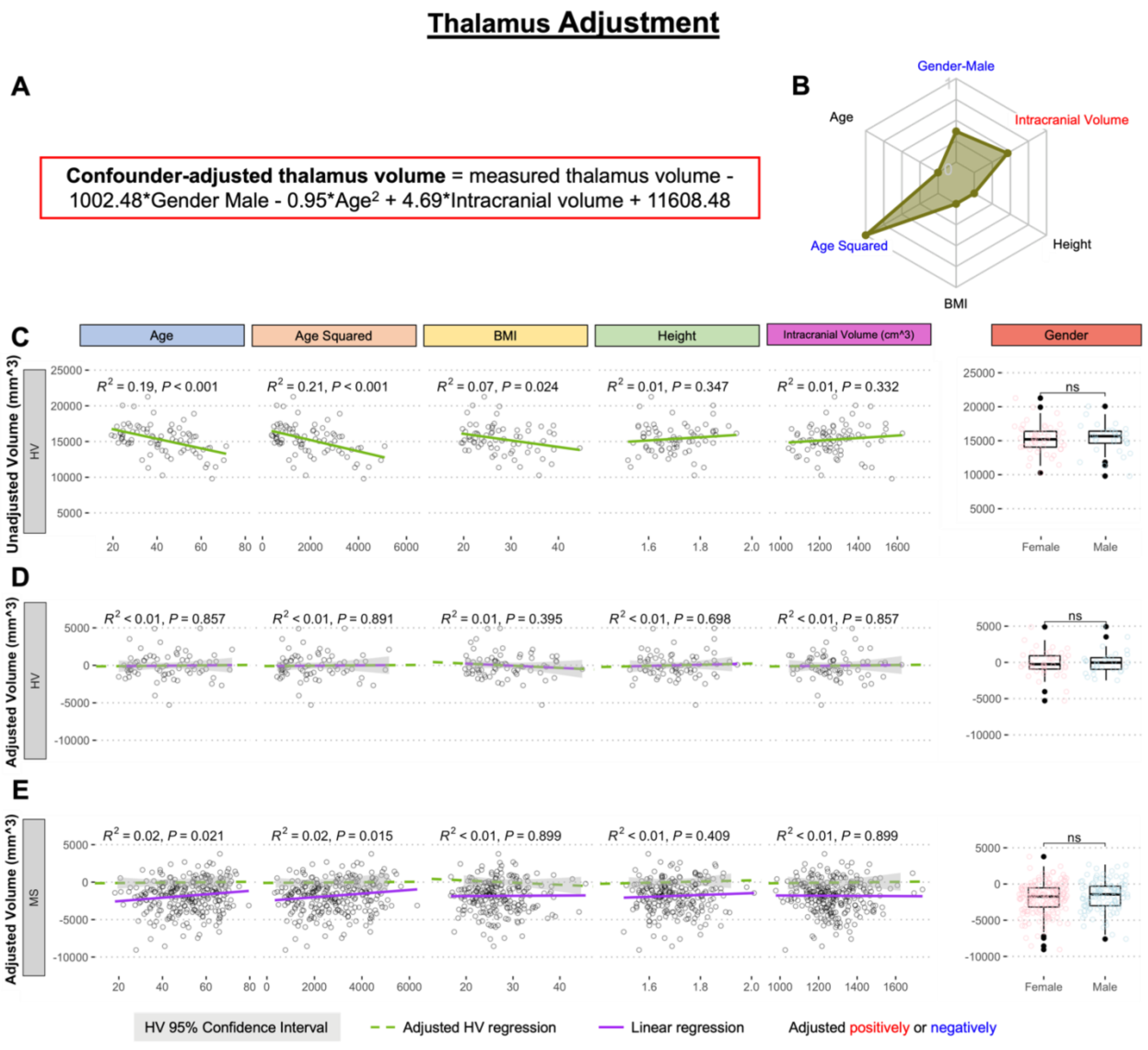

Figure S17. Adjustment of single MRI biomarker (i.e., thalamus volume) for six physiological confounders. A. The final equation for the confounder-adjusted thalamus volume is shown at the top in red outline. This equation was derived from multiple linear regression models as described in the Method section. **B.** In the top right corner is resulting radar chart that show proportional weights of the applied confounder adjustment, with confounders with lowest weights (i.e., the innermost circle) representing zeros. **C.** Univariate linear regression models between each tested confounder on x-axis (first 5 graphs) or gender (sixth graph) and measured

thalamus volume on the y-axis in 80 healthy volunteers (HV). **D.** Same univariate regressions in the HV cohort after applying adjustment formula show no remaining residual effect of confounders. **E.** Applying HV-derived adjustment formula to MS cohort shows no residual effect, when considering Bonferroni adjustment for multiple comparisons (i.e., $p<0.05/12 = p<0.004$).

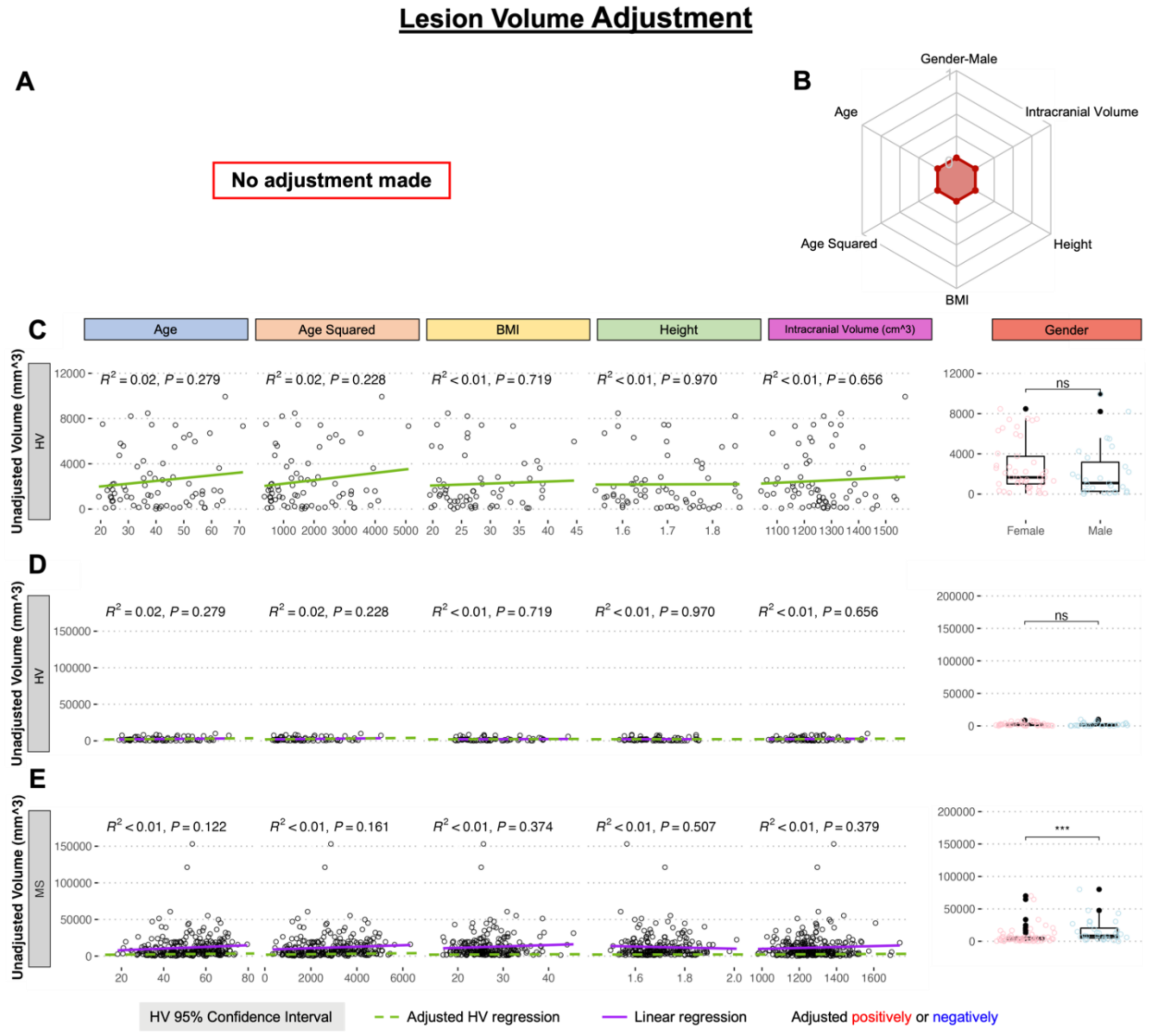

Figure S18. Adjustment of single MRI biomarker (i.e., lesion volume) for six physiological confounders. A. No confounder-adjustment was made for lesion volume. Possible adjustment was derived from multiple linear regression models as described in the Method section. **B.** In the top right corner is resulting radar chart that show proportional weights of the applied confounder adjustment, with confounders with lowest weights (i.e., the innermost circle) representing zeros. **C.** Univariate linear regression models between each tested confounder on x-axis (first 5 graphs) or gender (sixth graph) and measured lesion volume on the y-axis in 80 healthy volunteers (HV).

D. No univariate linear regression of confounders was seen in the HV cohort. **E.** No univariate linear regression of confounders was seen in the MS cohort.

Unadjusted

Scale	Feature	R Squared	Rho	p-value
CombiWISE	Age	0.211	0.440	1.18E-12
	Spinal Cord	0.161	-0.407	2.71E-10
	Ventricular CSF	0.104	0.338	1.27E-07
	Sulcal CSF	0.021	0.143	2.77E-02
	Cerebellum WM	0.041	-0.259	6.44E-05
	Supratentorial WM	0.027	-0.152	2.02E-02
	Normal-appearing WM	0.088	-0.255	7.66E-05
	Thalamus	0.125	-0.380	1.67E-09
	Caudate	0.045	-0.231	3.43E-04
	Brain Parenchymal Fraction	0.097	-0.300	2.55E-06
	Lesion Volume	0.028	0.346	4.75E-08
EDSS	Age	0.241	0.443	8.69E-13
	Spinal Cord	0.160	-0.408	2.48E-10
	Ventricular CSF	0.081	0.291	6.19E-06
	Sulcal CSF	0.012	0.112	8.54E-02
	Cerebellum WM	0.034	-0.226	4.98E-04
	Supratentorial WM	0.025	-0.121	6.69E-02
	Normal-appearing WM	0.073	-0.213	1.04E-03
	Thalamus	0.109	-0.321	4.76E-07
	Caudate	0.041	-0.183	4.72E-03
	Brain Parenchymal Fraction	0.071	-0.242	1.68E-04
	Lesion Volume	0.018	0.280	1.25E-05
NeurEx	Age	0.194	0.470	2.00E-14
	Spinal Cord	0.145	-0.404	3.73E-10
	Ventricular CSF	0.090	0.327	3.30E-07
	Sulcal CSF	0.014	0.121	6.30E-02
	Cerebellum WM	0.067	-0.296	4.12E-06
	Supratentorial WM	0.048	-0.224	5.93E-04
	Normal-appearing WM	0.099	-0.310	1.26E-06
	Thalamus	0.149	-0.414	3.43E-11
	Caudate	0.062	-0.265	3.60E-05
	Brain Parenchymal Fraction	0.090	-0.301	2.29E-06
	Lesion Volume	0.036	0.337	1.07E-07
SDMT	Age	0.123	-0.368	5.19E-09
	Spinal Cord	0.030	0.196	3.17E-03
	Ventricular CSF	0.220	-0.495	9.90E-16
	Sulcal CSF	0.071	-0.261	4.85E-05
	Cerebellum WM	0.059	0.301	2.93E-06
	Supratentorial WM	0.028	0.192	3.12E-03
	Normal-appearing WM	0.063	0.282	1.12E-05
	Thalamus	0.263	0.549	5.92E-20
	Caudate	0.198	0.465	4.02E-14
	Brain Parenchymal Fraction	0.217	0.468	2.66E-14
	Lesion Volume	0.142	-0.493	6.27E-16

Adjusted

Scale	Feature	R Squared	Rho	p-value
CombiWISE	Age	0.211	0.440	1.18E-12
	Spinal Cord	0.063	-0.254	1.22E-04
	Ventricular CSF	0.068	0.278	1.67E-05
	Sulcal CSF	0.000	0.013	8.46E-01
	Cerebellum WM	0.016	-0.147	2.46E-02
	Supratentorial WM	0.047	-0.203	1.90E-03
	Normal-appearing WM	0.130	-0.304	2.03E-06
	Thalamus	0.006	-0.094	1.49E-01
	Caudate	0.003	-0.059	3.67E-01
	Brain Parenchymal Fraction	0.008	-0.087	1.80E-01
	Lesion Volume	0.028	0.346	4.75E-08
EDSS	Age	0.241	0.443	8.69E-13
	Spinal Cord	0.057	-0.264	6.77E-05
	Ventricular CSF	0.045	0.223	6.08E-04
	Sulcal CSF	0.001	-0.042	5.21E-01
	Cerebellum WM	0.010	-0.119	7.08E-02
	Supratentorial WM	0.039	-0.163	1.27E-02
	Normal-appearing WM	0.102	-0.249	1.17E-04
	Thalamus	0.001	-0.036	5.79E-01
	Caudate	0.001	-0.011	8.72E-01
	Brain Parenchymal Fraction	0.001	-0.018	7.81E-01
	Lesion Volume	0.018	0.280	1.25E-05
NeurEx	Age	0.194	0.470	2.00E-14
	Spinal Cord	0.056	-0.240	3.06E-04
	Ventricular CSF	0.060	0.260	5.93E-05
	Sulcal CSF	0.001	0.008	9.07E-01
	Cerebellum WM	0.028	-0.169	9.64E-03
	Supratentorial WM	0.064	-0.270	3.16E-05
	Normal-appearing WM	0.121	-0.352	2.94E-08
	Thalamus	0.014	-0.111	8.83E-02
	Caudate	0.007	-0.074	2.60E-01
	Brain Parenchymal Fraction	0.011	-0.085	1.93E-01
	Lesion Volume	0.036	0.337	1.07E-07
SDMT	Age	0.123	-0.368	5.19E-09
	Spinal Cord	0.003	0.073	2.80E-01
	Ventricular CSF	0.194	-0.476	1.64E-14
	Sulcal CSF	0.038	-0.195	2.58E-03
	Cerebellum WM	0.039	0.238	2.47E-04
	Supratentorial WM	0.074	0.289	6.76E-06
	Normal-appearing WM	0.120	0.385	9.00E-10
	Thalamus	0.101	0.311	1.14E-06
	Caudate	0.107	0.335	1.26E-07
	Brain Parenchymal Fraction	0.093	0.296	3.56E-06
	Lesion Volume	0.142	-0.493	6.27E-16

Supplementary Table S1. Unilateral correlations of disability outcomes with MRI features.

The left panel shows R squared, Spearman correlation coefficients (Rho), and p-values between unadjusted MRI features and disability outcomes. The right panel shows R squared, Spearman correlation coefficients, and p-values between confounder-adjusted MRI features and disability outcomes.

Scale	Cohort	Model	RMSE	R Squared	Rho	CCC	p-value
CombiWISE	Training	Unadjusted	10.10	0.74	0.86	0.76	2.30E-63
		Adjusted	10.79	0.70	0.83	0.71	2.13E-54
	Training (Cross Validation)	Unadjusted	14.11	0.31	0.58	0.47	4.97E-03
		Adjusted	14.89	0.33	0.54	0.43	1.15E-02
	Validation	Unadjusted	17.57	0.21	0.48	0.34	2.56E-08
		Adjusted	17.01	0.26	0.52	0.35	1.89E-09
EDSS	Training	Unadjusted	1.20	0.71	0.80	0.72	9.83E-49
		Adjusted	1.21	0.69	0.78	0.71	3.84E-43
	Training (Cross Validation)	Unadjusted	1.71	0.33	0.48	0.44	2.62E-02
		Adjusted	1.67	0.29	0.50	0.45	2.08E-02
	Validation	Unadjusted	1.84	0.18	0.47	0.31	5.93E-08
		Adjusted	1.75	0.26	0.49	0.39	2.49E-08
NeurEx	Training	Unadjusted	50.69	0.69	0.84	0.73	1.83E-56
		Adjusted	51.60	0.69	0.84	0.72	2.93E-56
	Training (Cross Validation)	Unadjusted	71.47	0.36	0.58	0.49	6.35E-03
		Adjusted	69.98	0.32	0.58	0.46	5.54E-03
	Validation	Unadjusted	86.49	0.20	0.47	0.35	8.92E-08
		Adjusted	83.02	0.26	0.54	0.38	3.58E-10
SDMT	Training	Unadjusted	8.53	0.69	0.84	0.72	9.49E-59
		Adjusted	7.26	0.78	0.89	0.81	7.38E-75
	Training (Cross Validation)	Unadjusted	10.98	0.33	0.61	0.46	3.76E-03
		Adjusted	10.43	0.40	0.66	0.51	1.18E-03
	Validation	Unadjusted	12.26	0.33	0.57	0.47	2.23E-11
		Adjusted	12.16	0.34	0.57	0.49	2.93E-11

Supplementary Table S2. Statistics on model performances in each cohort.

This table shows model performances in predicting disability scales individually within the training cohort, training cohort with cross validation, and independent validation cohort.
